# An Adaptive Framework for Selecting Environmental Monitoring Protocols to Support Ocean Renewable Energy Development

**DOI:** 10.1100/2012/450685

**Published:** 2012-12-23

**Authors:** Emily J. Shumchenia, Sarah L. Smith, Jennifer McCann, Michelle Carnevale, Grover Fugate, Robert D. Kenney, John W. King, Peter Paton, Malia Schwartz, Malcolm Spaulding, Kristopher J. Winiarski

**Affiliations:** ^1^Graduate School of Oceanography, University of Rhode Island, South Ferry Road, Narragansett, RI 02882, USA; ^2^Coastal Resources Center and Rhode Island Sea Grant, University of Rhode Island, Narragansett, RI 02882, USA; ^3^Rhode Island Coastal Resources Management Council, South Kingstown, RI 02879, USA; ^4^College of the Environmental and Life Sciences, University of Rhode Island, Kingston, RI 02881, USA; ^5^College of Engineering, University of Rhode Island, Narragansett, RI 02882, USA

## Abstract

Offshore renewable energy developments (OREDs) are projected to become common in the United States over the next two decades. There are both a need and an opportunity to guide efforts to identify and track impacts to the marine ecosystem resulting from these installations. A monitoring framework and standardized protocols that can be applied to multiple types of ORED would streamline scientific study, management, and permitting at these sites. We propose an adaptive and reactive framework based on indicators of the likely changes to the marine ecosystem due to ORED. We developed decision trees to identify suites of impacts at two scales (demonstration and commercial) depending on energy (wind, tidal, and wave), structure (e.g., turbine), and foundation type (e.g., monopile). Impacts were categorized by ecosystem component (benthic habitat and resources, fish and fisheries, avian species, marine mammals, and sea turtles) and monitoring objectives were developed for each. We present a case study at a commercial-scale wind farm and develop a monitoring plan for this development that addresses both local and national environmental concerns. In addition, framework has provided a starting point for identifying global research needs and objectives for understanding of the potential effects of ORED on the marine environment.

## 1. Introduction

At present, there is a great need to better understand the potential effects of offshore renewable energy developments (ORED) on the marine environment [1–3]. While the development of commercial-scale ORED in the United States has lagged well behind development in Europe [4], construction on multiple projects is likely to begin within the next few years in U.S. waters [5]. Because of differences between Europe and the U.S. in terms of regulatory requirements, environmental settings, and species present at development sites [6], there is a considerable need for U.S.-specific guidance to ensure thorough data collection as ORED projects develop to evaluate effects and assess potential impacts. In this paper, we use the word “effect” to refer to a change in the environment without respect to magnitude or direction (i.e., moderate or severe, positive or negative [1]). When the effect is better characterized and a magnitude and direction can be assigned, we refer to it as an “impact.” A scientific framework exists for detecting and characterizing effects, but more work is needed in order to describe and assess impacts [1]. 

Data collection at ORED sites should be guided by a framework that identifies the unique impacts associated with ORED [7], then selects and helps implement a suite of standardized environmental monitoring protocols relevant to each development type. Standardized protocols improve impact assessment by following a single methodology at multiple sites, permitting comparison and aggregation of data [8]. Building a uniform database of environmental impacts will allow us to better refine our understanding of drivers and stressors acting at ORED sites, improve the Environmental Impact Statement (EIS) process, and refine monitoring requirements in the future as certain impacts are suggested to be either negligible or worthy of concern. This knowledge can be used to encourage development in areas where known impacts are expected to be minimal. In addition, reducing the existing uncertainty about environmental impacts related to ORED will likely ease public concern about development and therefore improve the siting process. Such a framework should also assist in answering regulatory questions about siting and scale by ensuring that relevant data are collected, therefore reducing uncertainty in decision making.

Monitoring an effect or an impact means that the monitoring protocol must be designed to measure change against some baseline condition or management objective [9, 10]. This change may be measured temporally, as in between seasons or years, before and after construction, or spatially, including measuring differences between an affected and a control area. Designing a study that can both provide conclusive evidence of an impact (or lack thereof) and separate this impact from the noise of seasonal or interannual environmental variability can be problematic [11]. Furthermore, not all of the potential impacts will be directly observable. For example, observations of underwater ORED structures will not likely be continuous. Therefore, in order to determine if a foundation has an impact on seabed structure, for example, discrete measurements of seabed volume will be made and compared through time. In this example, measured changes in seabed volume serve as a representative of the impact of the foundation on the seabed and in this way are considered to be an indicator of that impact. In general, indicators can be used as a means to quantitatively track change in the context of ecosystem-based management goals [12–14]. Developing indicators of change at ORED sites would be immensely helpful to impact assessments and, if developed across disciplines (e.g., biology, geology, physical oceanography), enable an overall assessment of the condition of the ecosystem. Building support for an indicator as a representative of an ecosystem attribute or function is an iterative process that can be conducted as monitoring data are collected at ORED sites. At first, where few data exist, qualitative “reference directions” can be used to track change [10]. As a database is built, changes can be quantified and thresholds can be identified relative to impacts at particular developments. In rare cases where the natural variability of a parameter has already been characterized, statistical tools may be used to determine appropriate thresholds or even the sampling protocols themselves (e.g., power analyses [15–17]). In most cases, however, very little is known about natural variability and environmental monitoring efforts will be measuring natural change commingled with the effects of ORED. A monitoring framework that considers all of these concerns is essential.

To address these concerns, an adaptive, rather than a static, monitoring framework for ORED is most appropriate. Firstly, there are many points of weakness in the general understanding of the impacts of ORED on marine resources that could greatly change monitoring needs and/or requirements. For example, the impacts of indirect effects (e.g., alteration to food webs) and wholly unanticipated effects are unknown [1]. Data regarding these points may only become available at a later stage of ORED maturity, but current monitoring protocols and regulations should be prepared in anticipation of these types of effects. Next is the current understanding of linkages between effects and indicators. We can agree conceptually that certain environmental/biological parameters are indicative of an ecosystem change, but in many cases we have no estimate of thresholds of concern for these parameters (e.g., how much of a reduction can occur in a bird population before mitigation needs to take place?). Just as experience in ecosystem-based fisheries management has helped propose appropriate thresholds for indicators of fisheries status [9], experience in managing OREDs will help clarify the assumptions made between effects and indicators. An adaptive framework is also essential in a field where new technologies are developing and emerging at a rapid pace.

In this paper we propose an adaptive monitoring framework based on indicators of the likely changes to the ecosystem due to ORED. We developed the framework to be used by offshore renewable energy developers and U.S. management and regulatory agencies in order to standardize the design and methodologies used to collect data at ORED sites. The framework and protocols were developed to be scientifically valid and easy to understand and follow by nonscientists. In order to do so, we reduced the complexity of impact assessment by developing decision-support tools that guide users towards monitoring objectives. As scientists, we were challenged with the task of maintaining scientific rigor in the framework while simultaneously acknowledging the pressures on management and regulatory agencies to encourage developments and keep costs low. The resulting framework is adaptive, flexible and can be implemented at any ORED site in U.S. waters.

## 2. Methods

Offshore renewable energy development is here defined as the construction and operation of one or more devices designed to harness power from the marine environment (wind, tidal, and wave power considered here) and includes any necessary infrastructure, including subsea cables, the vessels necessary to construct or install an ORED, and the footprint of a project. This paper considers the effects of ORED on the benthic habitat and resources, marine mammals, sea turtles, fish, and avian species. We also considered the effect of ORED on one human use—fishing activity—because of the inextricability of the effects on fishing activity from effects on fish themselves, and the resulting concerns of fishermen about potential effects on their livelihood. We examined renewable energy developments at two scales, 1 = “demonstration” and 2 = “commercial/multiple commercial.” At Scale 1, three or fewer devices are part of a “farm”; Scale 2 constitutes a farm or farms of around 100 devices and greater. 

A literature review was conducted of potential positive and negative environmental effects (see Appendix  A, supplementary material available online at doi:10.1100/2012/450685 for works cited) using the Programmatic Environmental Impact Statement (PEIS) for Alternative Energy Development developed by the Minerals Management Service in 2007 as a reference point [18]. Potential impacts were categorized by the five affected ecosystem components, the anticipated level of effect (minor, moderate, major), and the level of certainty (high, medium, low) at each scale of development and for each technology type within an ORED “impact matrix” (Supplementary Appendix B). The descriptions and thresholds for impact levels were derived from the definitions used in the PEIS [18]: minor—should not influence or have only small impacts on the affected resource, activity, or community; moderate—impacts could moderately influence the resource, activity, or community, generally or for particular species; major—impacts could significantly influence the resource, activity, or community, generally or for particular species. Here, we used the word “certainty” to refer to the amount of evidence available from studies conducted on a particular effect. High certainty indicates that there was a large body of literature documenting or studying an impact. It is important to note that “certainty” does not refer to the chance that an impact will occur. The chance of an impact occurring is more appropriately described as likelihood, a concept that was not addressed in this study. Therefore, where we describe an effect with a high certainty of major impact, this can be interpreted as “*if* the named effect occurs, *then* the magnitude of the impact on environment will be major.”

Suites of similar potential impacts were aggregated by energy resource, foundation type, and scale of development in order to define “Impact scenarios.” An Impact scenario is applicable to multiple development situations in order to distill and focus the monitoring and management actions required for ORED. Impact scenarios describe the major and moderate negative impacts of ORED on five ecosystem components—benthic habitat and resources, marine mammals, sea turtles, fish, and avian species.

We developed two types of decision trees to serve as decision-support tools, to help users determine which impacts are relevant when their development criteria are implemented, and to evaluate alternatives. The first decision tree, the “Impact decision tree,” determines the approximate magnitude of impacts from ORED on each ecosystem component considering three factors—energy type, foundation type, and development scale. The second type, “Component decision trees,” is a suite of finer-scale decision trees for each of the ecosystem components that determine which monitoring protocols are recommended given a more specific suite of characteristics related to the development type (e.g., stage of development). We took this approach because each ecosystem component experiences different levels of impact due to different drivers. For example, different foundation types would differentiate several types of effect for benthic habitat and resources but are not likely to do the same for avian species.

A chart-based/graphic format was rejected in favor of a text-based/key format. In the adopted format, the user answers a series of questions about the development project and is guided through the decision tree and toward an eventual “answer” based on the responses to the questions. For the Impact decision tree, the “answer” is an Impact scenario, an associated list of the ecosystem components that may experience major and moderate negative (i.e., adverse) impacts from ORED, a short description of the type of impacts, and an estimate of the certainty regarding these impacts. For each scenario, the lists of major negative potential impacts were ranked by proportion and magnitude of total impacts so that #1 reflects the component with the most negative impacts. To provide more detail on potential adverse impacts, all moderate impacts and levels of certainty are also provided for each scenario. The lists of moderate impacts are not prioritized or ranked and are listed as they appeared in the impact matrix (Supplementary Appendix B). 

For the Component decision trees, the “answer” is a list of monitoring objectives. At the University of Rhode Island we have developed a series of monitoring protocols to address each of these objectives. We also developed a case study in order to demonstrate how a manager or regulator may use these tools to develop an ORED monitoring plan. The case study consists of a Scale 2 wind-turbine farm composed of around 200 jacketed structures. We describe the resulting Impact scenario and list the major and moderate impacts that monitoring should address. To demonstrate the next step, we present an example monitoring protocol that addresses monitoring of benthic habitat and resources.

## 3. Results

From the renewable energy impact matrix, literature review, and expert judgment, we assembled a short list of the potential effects for each ecosystem component considered to be of greatest importance. For each effect, we propose an indicator that is recommended for use as a monitoring target (Table 1).

### 3.1. The Monitoring Framework

**Figure 1:**
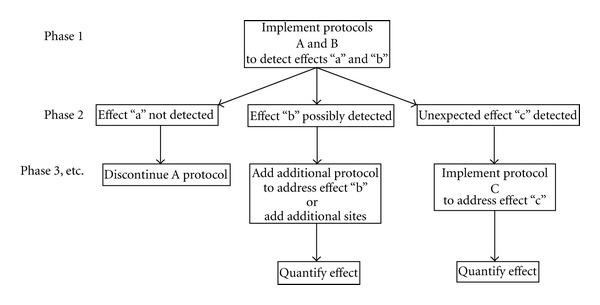
Proposed adaptive and reactive monitoring framework.

We developed an adaptive and reactive monitoring framework that incorporates the use of environmental indicators to track change (Figure 1). Currently, we have the ability to characterize a baseline condition and assign reference directions to indicators; for example, increases in sediment grain size at every turbine should accelerate monitoring for scour. Reference directions are useful when data are insufficient to establish more quantitative reference levels, but they only provide an indication of a trend and do not specify when a threshold of irreversible harm has been reached [10]. In an adaptive monitoring framework, data are synthesized to produce more quantitative metrics and thresholds for environmental indicators of ORED effects. In a reactive monitoring framework, evidence of an effect should be used to accelerate study of that effect, perhaps by multiple methodologies (refer to Figure 1). Suites of ORED effect indicators would not only provide a clearer path for goal setting for developers but would encourage regulatory monitoring protocols to contribute to our general understanding of the natural variation of marine ecosystems and how human activities can be integrated and harmonized.

### 3.2. Impact Decision Tree

The Impact decision tree determines the approximate magnitude of impacts from ORED on each ecosystem component for a broad range of development types and scenarios. For each combination, a bar graph shows the relative number of potential impacts and their magnitudes (Table 2). Even though there are 39 possible scenarios that result when combining the three factors, our decision tree reduces these to six main Impact scenarios.

### 3.3. Impact Scenarios

Impact scenarios are very brief descriptions of the major environmental concerns regarding categories of ORED with similar environmental impacts. Below, the Impact scenarios are accompanied by pie charts representing the total number of impacts for each scenario, categorized by whether the impact is positive (blue), minor negative (green), moderate negative (yellow), or major negative (red). Each of these sections of the pie is further broken down by ecosystem component.

**Figure 2:**
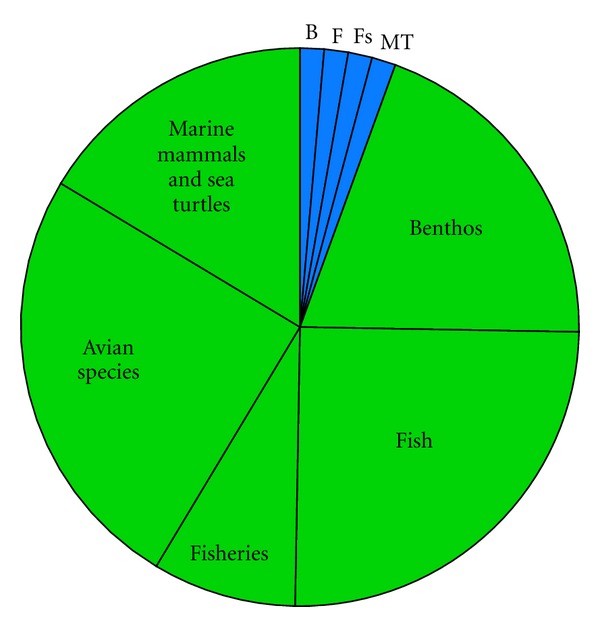
Pie chart representing the proportion of impacts for each ecosystem component for Impact scenario i1, demonstration-scale projects, categorized by whether the effect is positive (blue), minor negative (green), moderate negative (yellow), or major negative (red). B = Benthic habitat and resources; F = Fish; Fs = Fisheries; A = Avian species; MT = Marine mammals and sea turtles.

**(i1) All Demonstration Scale Projects (Figure 2, Table 3)**

These projects are described as “Scale 1”. The current literature suggests that any renewable energy development, if completed at the demonstration scale, will not have moderate or major impacts on the ecosystem components examined here. Therefore, we list the potential minor impacts and their certainty in the Impact decision tree. Of the suite of minor impacts, benthic habitat and resources, avian species, and fish species share an equally high proportion. Across ecosystem components, impacts with the highest certainty tend to be physical and chemical disturbances, such as disturbance from device installation, attraction to devices, or chemical spills. Impacts with low certainty include noise (except for marine mammals and sea turtles where the certainty for this impact is high), changes to energy regimes, and changes in organism energetic expense. Electromagnetic field (EMF) impact is the only impact that has low certainty consistently across all ecosystem components. Only those potential impacts with high certainty are listed in the decision tree; where certainty is low, it may be impossible to detect any impact at this magnitude.

**Figure 3:**
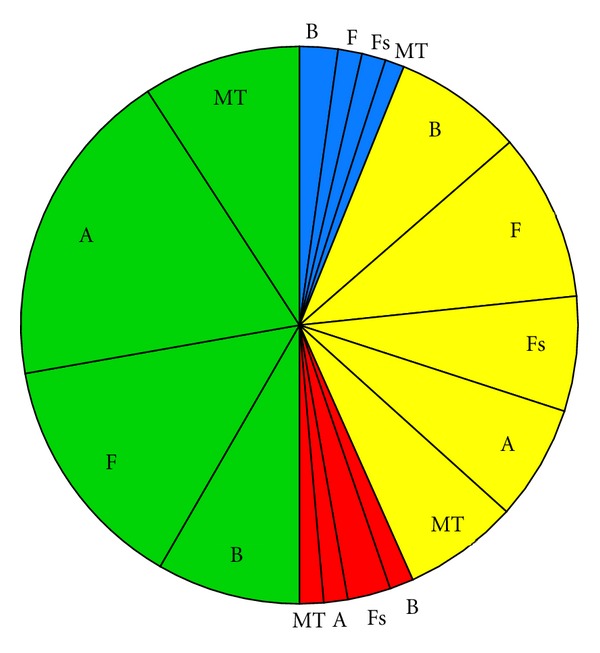
Pie chart representing the proportion of impacts for each ecosystem component for Impact scenario i2, wind turbine projects involving pile driving, categorized by whether the effect is positive (blue), minor negative (green), moderate negative (yellow), or major negative (red). B = Benthic habitat and resources; F = Fish; Fs = Fisheries; A = Avian species; MT = Marine mammals and sea turtles.

**(i2) Wind Turbine Developments Involving Pile Driving (Figure 3, Table 4)**

This scenario includes monopile wind turbine developments and jacketed- or tripod-mounted turbines at development Scale 2. If the proposed development will not utilize pile driving to install the jacketed or tripod structures, then Impact scenario i3 is more appropriate. The impacts that make this scenario unique are the presence of turbines above the water surface, the piles drilled into the seabed, and the noise associated with this activity. Therefore, the expected major impacts include noise, scour and/or deposition around the structures, displacement or attraction to structures, and loss of access to mobile-gear fishing grounds. Notable moderate impacts include resuspension of pollutants, loss of access to recreational and fixed gear fishing grounds, decreased catchability (fisheries), damaged/lost fishing gear, and collisions and strikes for avian species, marine mammals, and sea turtles. Reef effects are likely for benthic habitat and resources and fish species at these developments.

**Figure 4:**
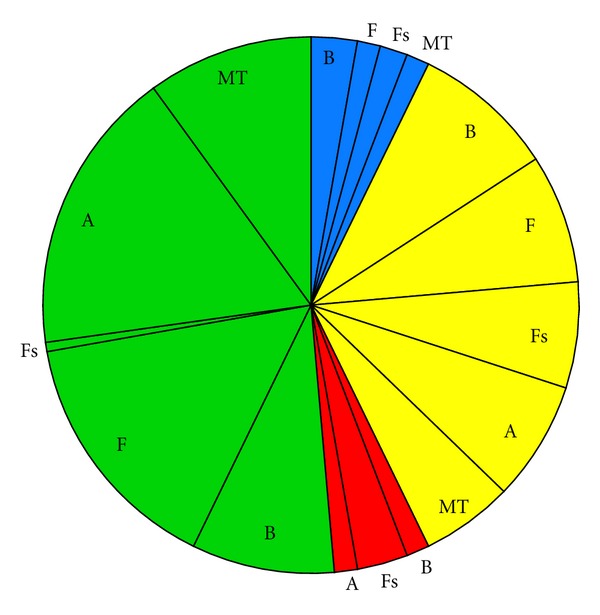
Pie chart representing the proportion of impacts for each ecosystem component for Impact scenario i3, wind turbine project involving no pile driving, categorized by whether the effect is positive (blue), minor negative (green), moderate negative (yellow), or major negative (red). B = Benthic habitat and resources; F = Fish; Fs = Fisheries; A = Avian species; MT = Marine mammals and sea turtles.

**(i3) Wind Turbine Developments Involving No Pile Driving (Figure 4, Table 5)**

Floating mooring or gravity-base foundations present a different suite of impacts for wind turbine developments at Scale 2. A major impact in scenario i2—noise during construction—is now absent. The suite of remaining negative impacts for each ecosystem component is very similar to i2, with the exception of benthic habitat and resources. Gravity-base foundations incur a moderate negative impact through physical disturbance to the sediment, where in i2 this impact is classified as minor. Reef effects are likely for benthic habitat and resources and fish species at these developments.

**Figure 5:**
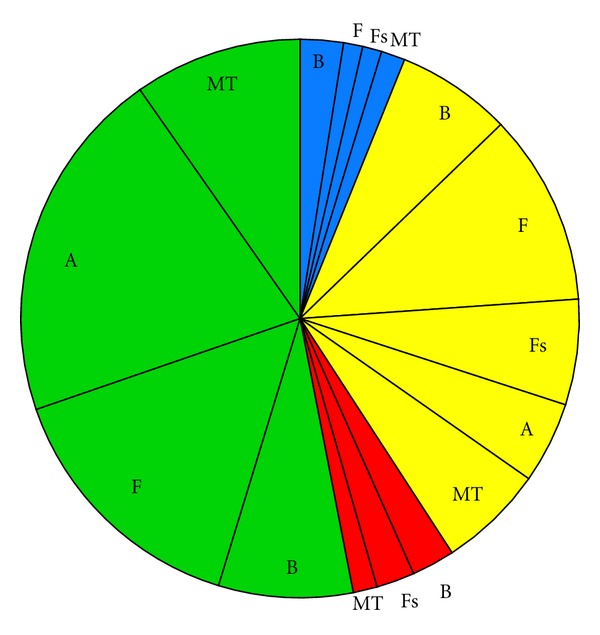
Pie chart representing the proportion of impacts for each ecosystem component for Impact scenario i4, bottom-mounted tidal turbine projects, categorized by whether the effect is positive (blue), minor negative (green), moderate negative (yellow), or major negative (red). B = Benthic habitat and resources; F = Fish; Fs = Fisheries; A = Avian species; MT = Marine mammals and sea turtles.

**(i4) Bottom-Mounted Tidal Turbine Projects (Figure 5, Table 6)**

For tidal turbine developments, the profile of impacts tended to differ more based on the foundation type than on whether the rotor is shrouded or open. Potential major impacts at these developments include changes to hydrodynamics, scour and/or deposition around devices/moorings, loss of access to mobile-gear fishing grounds, and noise from pile driving. If the proposed development will not utilize pile driving to install the tidal turbines, then Impact scenario i5 is more appropriate. Notable moderate impacts include physical disturbance to the sediment; collisions/strikes to rotor blades for fish species, avian species, marine mammals, and sea turtles; the effects of rotor wake/pressure gradients to fish and avian species; collisions/strikes with construction or support vehicles for marine mammals and sea turtles; decreased catchability and damaged/lost gear for fisheries.

**Figure 6:**
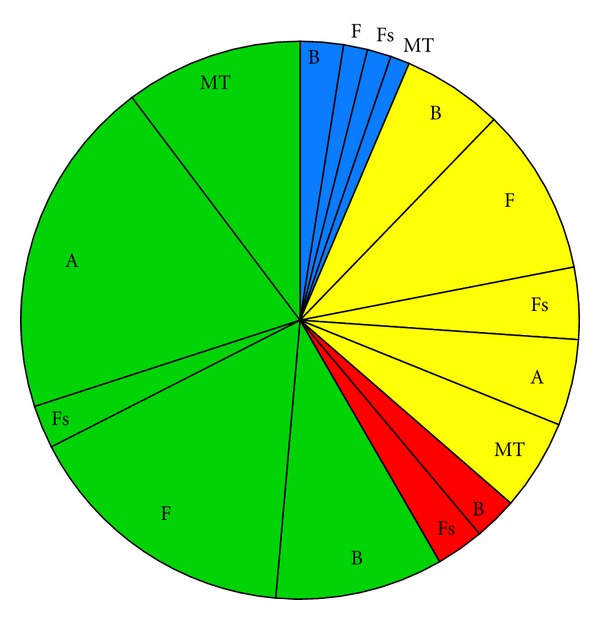
Pie chart representing the proportion of impacts for each ecosystem component for Impact scenario i5, floating mooring tidal turbine projects, categorized by whether the effect is positive (blue), minor negative (green), moderate negative (yellow), or major negative (red). B = Benthic habitat and resources; F = Fish; Fs = Fisheries; A = Avian species; MT = Marine mammals and sea turtles.

**(i5) Floating Mooring Tidal Turbine Projects (Figure 6, Table 7)**

Floating mooring foundations present a different suite of impacts for tidal turbine developments at Scale 2. A major impact in scenario i4—noise during construction—is now absent. The suite of remaining negative impacts for each ecosystem component is very similar to i4 with exceptions for benthic habitat and resources and fisheries. The impacts from sediment disturbance in this scenario are downgraded to minor, as are the impacts surrounding decreased catchability.

**Figure 7:**
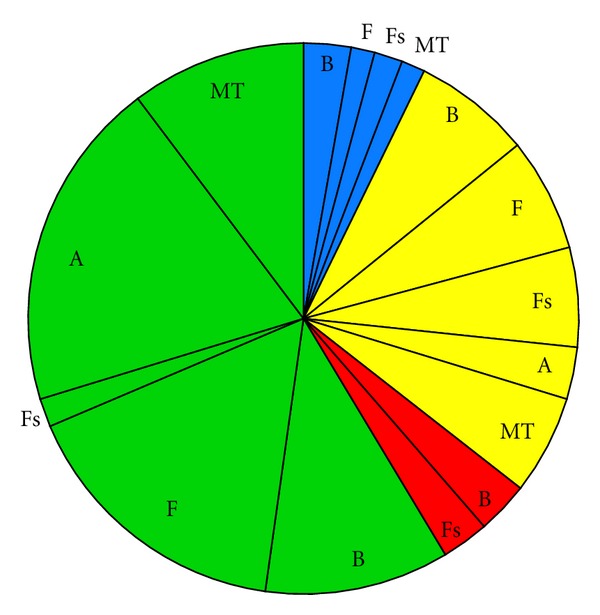
Pie chart representing the proportion of impacts for each ecosystem component for Impact scenario i6, wave energy project, categorized by whether the effect is positive (blue), minor negative (green), moderate negative (yellow), or major negative (red). B = Benthic habitat and resources; F = Fish; Fs = Fisheries; A = Avian species; MT = Marine mammals and sea turtles.

**(i6) Wave Energy Projects (Figure 7, Table 8)**

In general, wave energy developments are not as well studied as tidal or wind developments. Therefore, we caution against the interpretation that the pie chart suggests that wave energy developments have a lower proportion of potential major and moderate impacts than any other development type. Major impacts at Scale 2 wave energy projects are changes in hydrodynamics, scour, and/or deposition around devices and loss of access to mobile-gear fishing grounds. Notable moderate impacts include loss of access to fixed-gear and recreational fishing grounds, damaged/lost fishing gear, chemical spills, and collisions/strikes with construction or support vehicles for marine mammals and sea turtles. Specifically, oscillating wave surge converters have higher potential impacts over the other types (moderate versus minor) for operational noise on fish species, marine mammals, and sea turtles, and for sediment disturbance on benthic habitat and resources. Overtopping devices pose increased potential impacts over other types (moderate versus minor) on avian species for displacement or attraction to the device because of the above-water structure.

### 3.4. Component Decision Trees

The Component decision trees take component-specific concerns into consideration and terminate with a manageable number of recommended monitoring protocols and templates (Tables 9, 10, 11, 12, and 13). For example, the benthic environment decision tree (Table 9) describes 24 total monitoring scenarios but condenses them into a maximum of four monitoring templates. We have two separate decision trees for marine mammals and sea turtles, but one set of monitoring protocols, as in many cases a single protocol can be used to monitor both components. After working through the Impact decision tree, the user should then select the recommended Component decision trees in order to determine specifically which monitoring objectives will apply to that technology type.

### 3.5. Monitoring Protocols

The Component decision trees terminate with a total of 30 monitoring objectives across all ecosystem components. Monitoring protocols have been developed to address each of these objectives at the University of Rhode Island, but for brevity, the benthic habitat and resources monitoring protocol for seabed scour and/or deposition is presented.

## 4. Case Study: Commercial Wind Farm

This test case was conducted using a hypothetical wind farm in the Wind Energy Area defined by BOEM in federal waters off the Massachusetts and Rhode Island coasts. A wind farm being planned for this area may include around 200 turbines with jacketed structures. Ecological concerns for this development include the sensitivities of local bird populations (scoters, red-throated loons), important commercial fisheries (demersal fish, lobsters), and the occasional presence of endangered marine mammals (particularly North Atlantic right whales).

### 4.1. Potential Impacts and Monitoring Plan

The Impact decision tree identified this installation as an i2 Impact scenario (Table 4). We recommend that monitoring plans for the four impacts listed as potentially major and negative (Loss of access to grounds for commercial mobile-gear fishermen during construction and operation, displacement or attraction to a device for avian species, seabed scour and/or deposition, and noise from pile driving for marine mammals) be required by federal permitting organizations. An additional 24 impacts were identified as potentially moderate and negative; a subset of these should be considered as part of the permitting requirements but could also serve to inform additional monitoring that might be conducted by other federal or state agencies to address local environmental or stakeholder concerns. 

By working through the Component decision trees, we identified nineteen monitoring objectives/protocols that are applicable to this development (Table 14), demonstrating that protocols can be developed to track single and multiple impacts. For example, a sampling protocol for benthic community analysis can also be used to collect information about sediment grain size. Based on the magnitudes of impacts and the local concerns for this wind farm, we identified a subset of eight monitoring objectives/protocols that should be implemented by developers, federal agencies, or both (Table 14). These include monitoring for seabed scour and/or deposition; ventless trap surveys for lobster and trawl surveys for demersal fish; an examination of the spatial use of fishing activity; aerial surveys using both high-definition video and still photography for avian monitoring; and visual surveys, passive acoustic monitoring, and marine mammal observers to track impacts to marine mammals and sea turtles. At a larger (regional) scale, or in an area where perhaps less is known about the local biological resources, a monitoring plan could begin with all of the recommended objectives/protocols and gradually decrease this effort through time as protocols fail to be relevant or detect ecosystem change.

### 4.2. Example Monitoring Protocol

Finally, we present an example protocol to address seabed scour and/or deposition (Table 15). Similar protocols for each impact should be developed to implement a consistent and robust monitoring plan. Protocols such as this are intended to provide guidance to regulators and developers on the most suitable methods for detecting the indicators of impacts but, like the overall framework, are designed to be adaptive to regulatory needs and site-specific concerns. In our example, we provided estimates of cost for two different monitoring strategies so that monitoring activities might be prioritized based on ecological and financial factors. We recommend that similar protocols be developed based on best practices identified in the literature and do not aim to be definitive or to provide comprehensive lists of all available methodologies.

## 5. Discussion

This paper presents a system for selecting both the priority impacts of ORED to be monitored and the appropriate monitoring protocols to address these impacts for a given project. One of the lessons learned from this effort was the importance of a monitoring program that is adaptive to both regulatory needs and local concerns (e.g., [19]). In drafting a set of monitoring objectives we attempted to account for variability in regions, target species, and so forth, but decisions about the most appropriate ways to monitor ORED will still have to be made on a case-by-case basis. Drivers, impacts, indicators, and technologies available to use in a monitoring program are all expected to be site specific. Our framework is flexible enough to address these concerns and to be useful for developing monitoring plans on scales ranging from local to national.

### 5.1. Development of Monitoring Protocols

It would be impractical to monitor every interaction that could potentially result in an effect on an organism or abiotic component of the ecosystem. Protocols should target priority impacts for monitoring given the ecological or societal importance of the resource, activity, or community and the certainty of their magnitude of impact. Monitoring protocols should test a particular regulatory question that is linked to one or more of these potential effects. In this study, we assumed that regulators would want and need to focus on the major negative potential impacts of ORED. However, we identified a number of potential positive impacts that are also worthy of research and monitoring. The positive impacts, such as reef effects, should be considered in any impact assessment for the potential value they may provide to the wider marine environment [2].

Protocols may need to be developed with the assumption that there are no or insufficient existing data on the relevant species to establish reference levels prior to monitoring. In some cases, the data may exist but not at a scale appropriate for integration into monitoring efforts. In other circumstances, baseline data will exist that can and should be incorporated into monitoring efforts. As described above, we assign reference directions to indicators when we have insufficient data to establish a quantitative threshold. Protocols should therefore be flexible to incorporate new and existing data for better estimates of particular reference points. Where there is an ongoing environmental monitoring program in the project area, and if the methods in use are sufficient to detect a change due to development, data collection should continue using the same methodology for comparable data. Examples of existing monitoring programs with appropriate data for incorporating into ORED studies may include species monitored under the Endangered Species or Marine Mammal Protection Acts for which monitoring may be occurring as part of a stock assessment program. Monitoring data collected for threatened, endangered, or other protected species could be compared directly to existing reference levels. For these species in particular it will be important to know if negative impacts are caused by ORED of any kind because such impacts will trigger immediate federal regulatory response/mitigation measures. In many cases, site-specific monitoring will still be desirable to analyze change related directly to the ORED.

The time scales of monitoring protocols should be long enough to observe short-term or immediate impacts caused by an ORED, include enough data to limit some of the effects of natural variability on the analysis, and last long enough to observe whether conditions return to a preconstruction state. Developer-led monitoring will probably not be conducted on time scales of a length sufficient to observe very long-term effects from ORED (i.e., decades). Supplementary monitoring should be conducted for a decade or more in order to understand long-term effects. For example, five years of monitoring may be enough time to observe effects on some species but may not be sufficient to identify stock- or population-level effects, particularly on slow-growing or long-lived species such as elasmobranchs. Additionally, some have speculated that if offshore renewable energy devices result in reef effects, this could create secondary effects such as larval spillover if spawning is occurring around the devices. These sorts of secondary effects may not be observable during the time scales of developer-led monitoring. Thus we recommend that, where feasible, monitoring and supplementary studies take place well beyond the minimum time frames required by federal permitting agencies.

### 5.2. Demonstration-Scale Projects as Opportunities

Demonstration-scale projects provide an opportunity for research to reduce some of the existing uncertainty around the potential environmental effects of offshore renewable energy projects, assisting regulators in prioritizing monitoring needs and making better decisions. Due to their size, individual demonstration-scale projects should be considered separately from commercial-scale projects in the extent to which monitoring should be required. Demonstration-scale projects are not expected to result in environmental impacts of the same magnitudes as commercial projects for any of the renewable energy device types. These projects, or those testing new technologies, should however be subject to more extensive monitoring relative to the scale of the potential impact, at least in the early phases of these technologies, due to the high levels of uncertainty surrounding impacts. Greater monitoring effort at these early stages may later reduce monitoring requirements at commercial-scale facilities, as impacts are better understood. We recommend that the monitoring requirements for demonstration-scale projects be adaptive; more studies that respond to the major and moderate impacts of a commercial-scale project should also be conducted initially as these projects are deployed, particularly at a stage where there are few or no commercial-scale facilities available for monitoring. As impacts are better characterized and methodologies are made more efficient, individual monitoring activities could be phased down in order to maximize the suite of activities at each development. Overall, we recommend that as many monitoring protocols be implemented as is feasible for the early stages of ORED development in the U.S.

### 5.3. Multiple Projects and Cumulative Impacts

Without a more complete understanding of the direct impacts of ORED on the various environment components discussed here, it is infeasible to develop a monitoring framework to address multiple projects or cumulative impacts. While the likelihood of an effect at the stock or population scale may increase with multiple ORED projects in a given area, not enough conclusive evidence exists at this point to indicate whether those effects are additive or increase in a nonlinear fashion. At the stage in which there are multiple projects in an area, monitoring may need to occur on a regional scale to understand the magnitude of an impact and may need to occur over a longer time series, and data collected from separate projects may need to be analyzed together to more completely understand what is happening. Meta-type analyses could be considered to attempt to maximize the utility of data collected from multiple projects.

As monitoring data are collected at single projects and analyzed, the potential effects at the individual project level will become better understood, and some of these impacts may be found to be negligible. However, in some cases an impact could be negligible when there is a single project but more severe when combined from multiple projects. It is not known *a priori *which situation is applicable to a particular project-species interaction.

### 5.4. Effects and Indicators

Currently there is a great deal of uncertainty surrounding the environmental effects of offshore renewable energy technologies. Through implementing a comprehensive monitoring program at each new ORED and by comparing and aggregating data, much of this uncertainty will be reduced and negative impacts will be better understood. Additional studies, including both *in situ* and laboratory-based research, are needed to better understand ORED drivers of ecosystem change and impacts. In many cases this is beyond the scope of what can be determined by a straightforward monitoring study and may be unreasonable to require of a developer. Regardless, it would be best if studies on these unknowns are ongoing and occur alongside other monitoring efforts. Paradoxically, without a better understanding of these impacts, it may not be feasible to design a protocol to be applied on a widespread scale.

Our work has provided a starting point for identifying these types of research priorities. Those effects found to have a low level of certainty are those for which additional research should be conducted. We recommend research funding and effort be directed toward understanding the level of risk of the following potential effects.

Benthic habitat and resources:

- changes to currents or wave regimes,
- increase in sediment temperature around cable,
- reef effects on marine hydrokinetic (MHK) devices,
- noise effects from construction, operation, and decommissioning,
- EMF effects from the power cables.

Fish species and fisheries:

- effect of pressure and velocity gradients around a rotor, and rotor wake, for tidal devices,
- effects resulting from chemical discharge, including leaking, spills, or flaking of marine coating,
- noise effects, including preconstruction noise from seismic surveying, construction noise, vessel noise, and operational noise,
- EMF effects from the power cables,
- changes to community composition from reef effects or disturbance,
- changes in species distribution.

Avian species:

- collision with rotating turbine blades from tidal devices,
- pressure and velocity gradients around a rotor for both tidal and wind turbines,
- EMF effects from the power cables,
- changes to foraging due to changes in turbulent dissipation/boundary layers for MHK devices,
- changes to foraging due to changes in the wave energy regime, for all device types.

Marine mammals and sea turtles:

- reef effects from devices,
- entanglement with mooring lines or cables,
- potential for effects from diffusion or flaking of marine coating,
- effects of operational noise, especially from tidal and wave energy devices,
- EMF effects from the power cables.

The potential for effects from EMF on all species has emerged as a particular issue of concern; there is considerable uncertainty around what the effects of EMF might be on each of the topic areas considered in this paper. When possible, we recommend site-based EMF studies be conducted alongside other monitoring projects.

## 6. Conclusions

The tools developed for this project represent an important first step in standardizing monitoring for offshore renewable energy projects in the United States. The data collected through a standardized monitoring program will provide a means for refining our understanding of the potential effects of offshore renewable energy projects and will feed back into better siting decisions. It is our hope that these results will prove useful to scientists, developers, managers, and regulators in understanding environmental changes resulting from offshore renewable energy development.

## Supplementary Material

The online supplementary material consists of a list of works cited from a literature review of the environmental impacts of renewable energy developments (Appendix A) and a series of tables that summarize the major impacts on multiple ecosystem components at two scales (Appendix B). We grouped impacts by ecosystem component: benthic habitat and resources, fish and fisheries resources, avian species, marine mammals and sea turtles. We further divided impacts by scale: “scale 1” represents demonstration-scale developments consisting of fewer than three devices and “scale 2” represents commercial-scale developments consisting of 100 or more devices. Finally, we differentiate impacts based on renewable energy device and foundation type. This impact assessment provided the foundation for developing decision support tools based on affected ecosystem component, scale, and device type.

## Figures and Tables

**Table 1:** Potential effects of offshore renewable energy developments in the United States considered to be of greatest importance based on results of a literature review and expert judgment (see Supplementary Appendix A). Effects are organized by ecosystem component and are paired with a proposed indicator of that effect.

	Impact/monitoring objective	Indicator
Benthic habitat and resources	Changes to seafloor morphology and structure (compared to preconstruction)	Increase or decrease in seabed volume
	Changes in median grain size, or organic content	(i) Deposition: decrease in median grain size, increase in organic content, increase in seabed volume (ii) Scour: increase in median grain size, decrease in organic content, decrease in seabed volume
	Turbidity during construction/decommissioning	Change in water column turbidity
	Change in target species abundance and distribution (e.g., species of importance)	Change in abundance, diversity, % cover, multivariate community composition
	Current speed/direction inside and outside farm	Change in residual flow rates
	Reef effects, colonization on foundations	Increase in % cover, biomass of epifaunal organisms; increase in presence of nonnative species
	Change in density, diversity, dominance structure of infauna	Change in abundance, diversity, % cover, multivariate community composition

Fish	Reef or aggregation effects	Increase in fish abundance around devices, shift in species composition, increase in presence of nonnative species
	Changes to abundance/distribution caused by disturbance or habitat alteration	Increase or decrease in fish abundance; increase or decrease in target species; shift in species composition; change in density, diversity, and dominance structure of fish species; increase in presence of nonnative species
	Blade strikes/pressure gradients (tidal power)	Observation of blade strike incidents
	EMF effects	Not feasible to monitor directly—changes in fish abundance, behavior, or species composition are indicators
	Installation or operational noise effects	Not feasible to monitor directly—changes in fish abundance, behavior, or species composition are indicators

Fisheries	Catchability (catch per unit effort) during construction	Catch per unit effort increases or decreases for target species
	Catchability (catch per unit effort) during operation	Catch per unit effort increases or decreases for target species
	Loss of access to grounds	Changes in numbers of vessels fishing near or inside of the renewable energy area, change in the presence of fixed fishing gear inside of or around a renewable energy installation
	Changes in species distribution	Shift in species composition, increase in presence of nonnative species
	Reef effects (aggregation)	Increase in fish abundance around devices; shift in species composition; increase in presence of nonnative species

Avian	Displacement/attraction	Increase or decrease in avian species-specific densities postconstruction in development area
	Barrier effects—effects on foraging, roosting, migratory movements	Migrating or commuting birds avoiding developed areas
	Collision mortality	Birds found dead or injured due to direct collision with infrastructure above the water

Marine mammals and sea turtles	Vessel strikes	Detection of dead or injured animals
	Noise generated during construction	Detection of dead or injured animals; changes in distribution, abundance, or behavior of populations
	Disturbance or injury during all stages of development, including from vessels	Detection of dead or injured animals; changes in distribution, abundance, or behavior of populations
	Noise generated during operation	Changes in distribution, abundance, or behavior of populations

**Table 2:** The impact decision tree. Each step of the key is followed until an Impact scenario is reached, denoted by “i#”. A bar graph is shown with each Impact scenario displaying the proportion of impacts, color-coded by direction (positive or negative) and magnitude (none, minor, moderate major). Data is derived from the literature review (Supplementary Appendix A) and renewable energy impact matrix (Supplementary Appendix B).

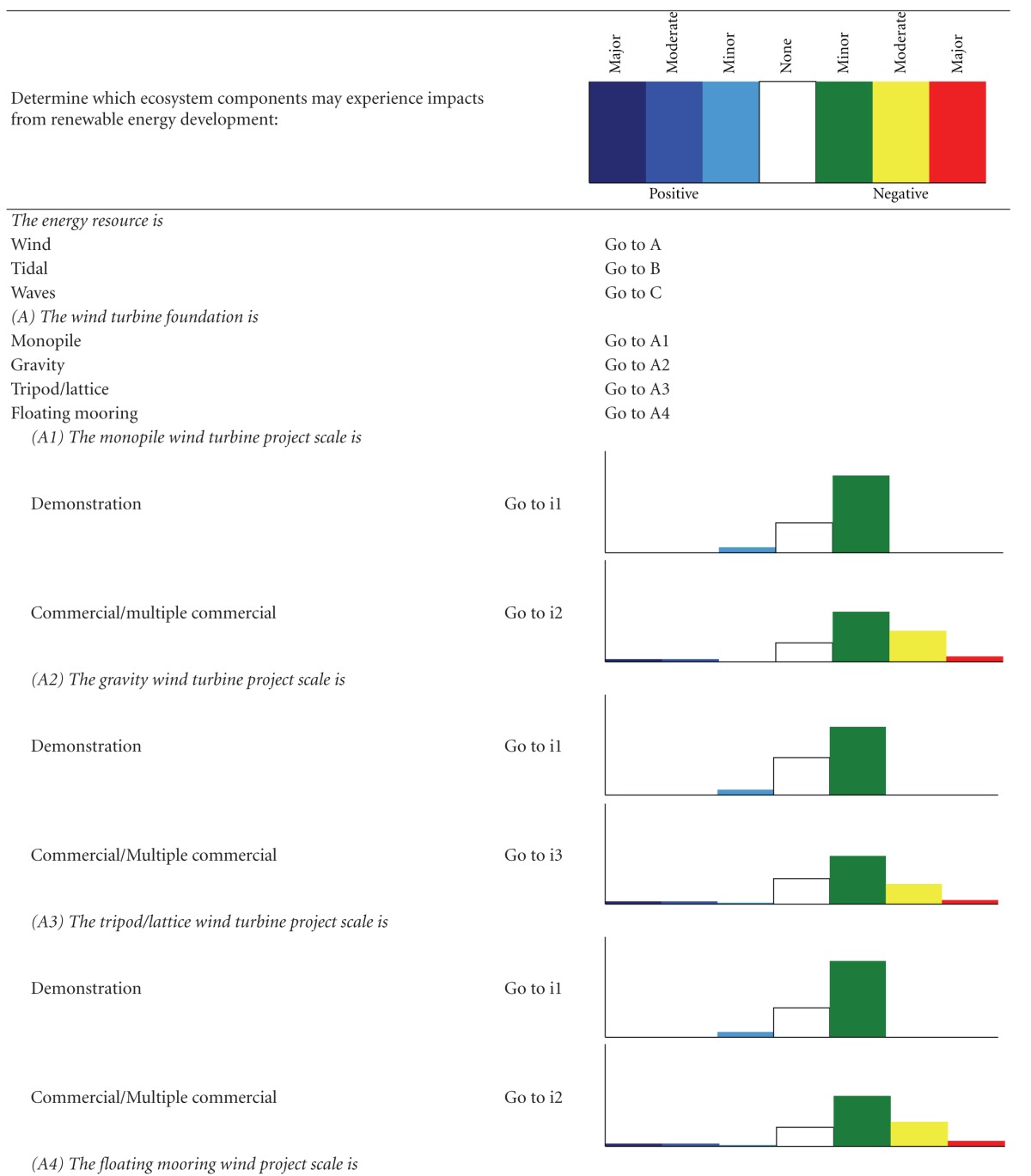 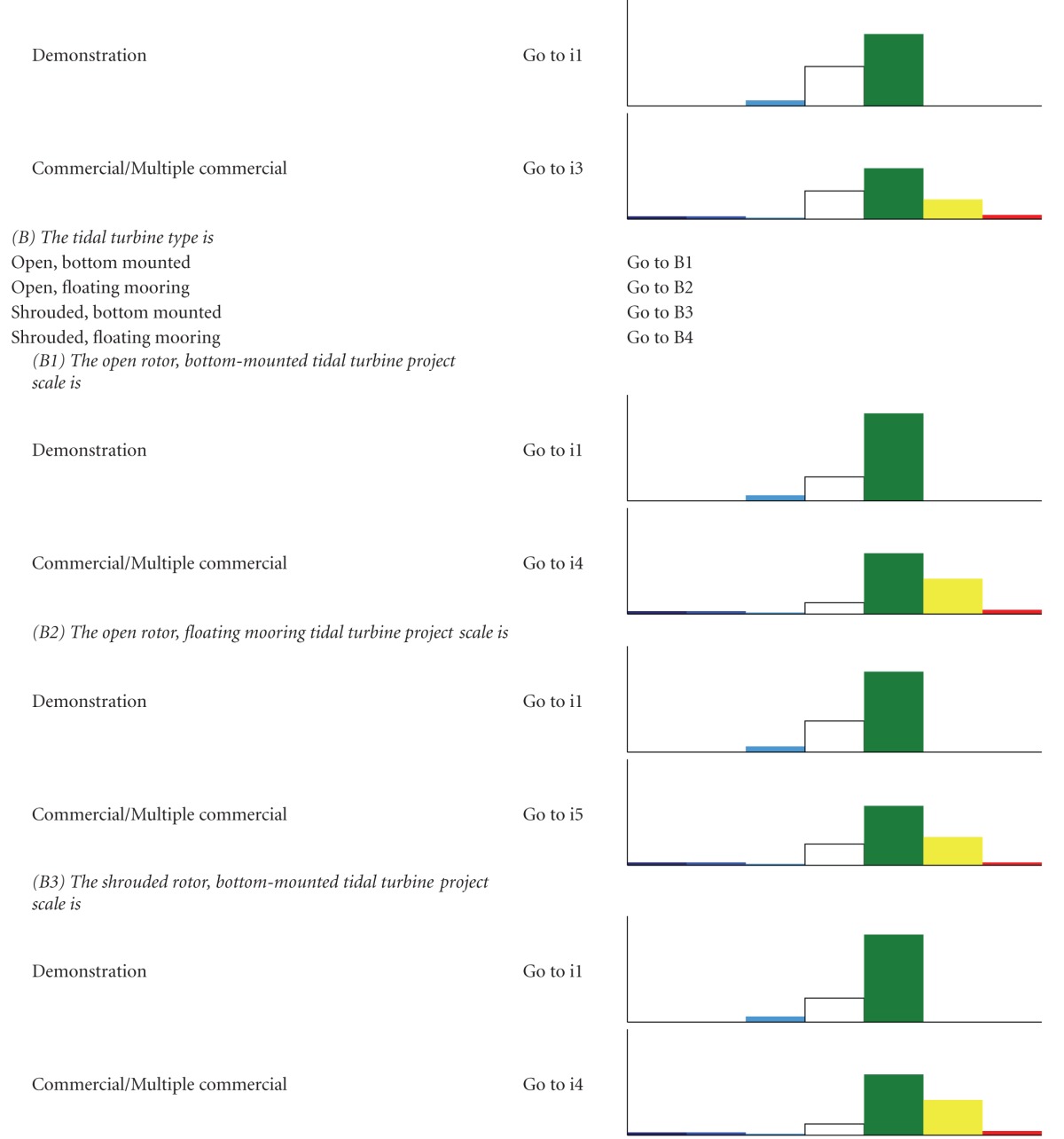 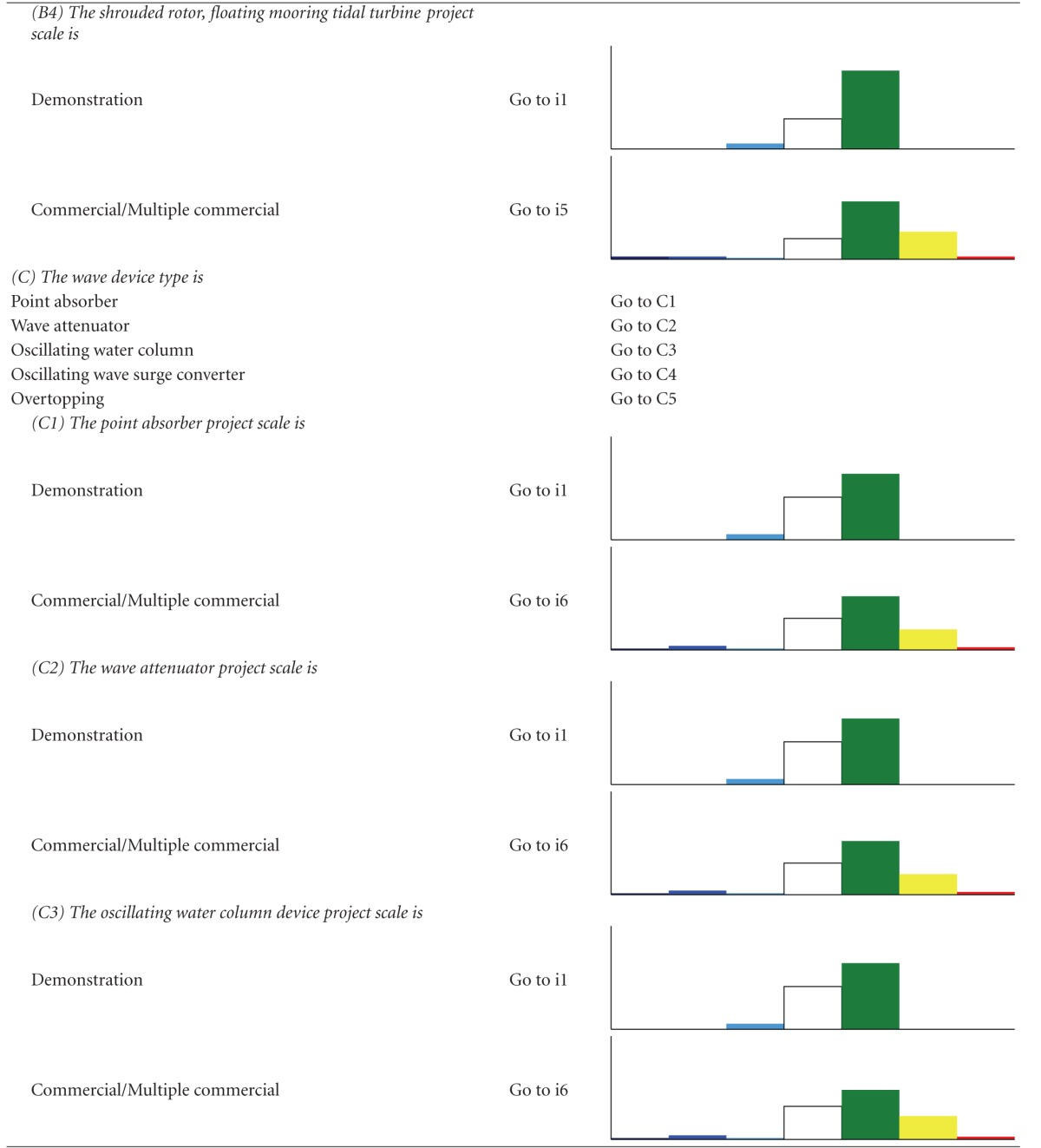 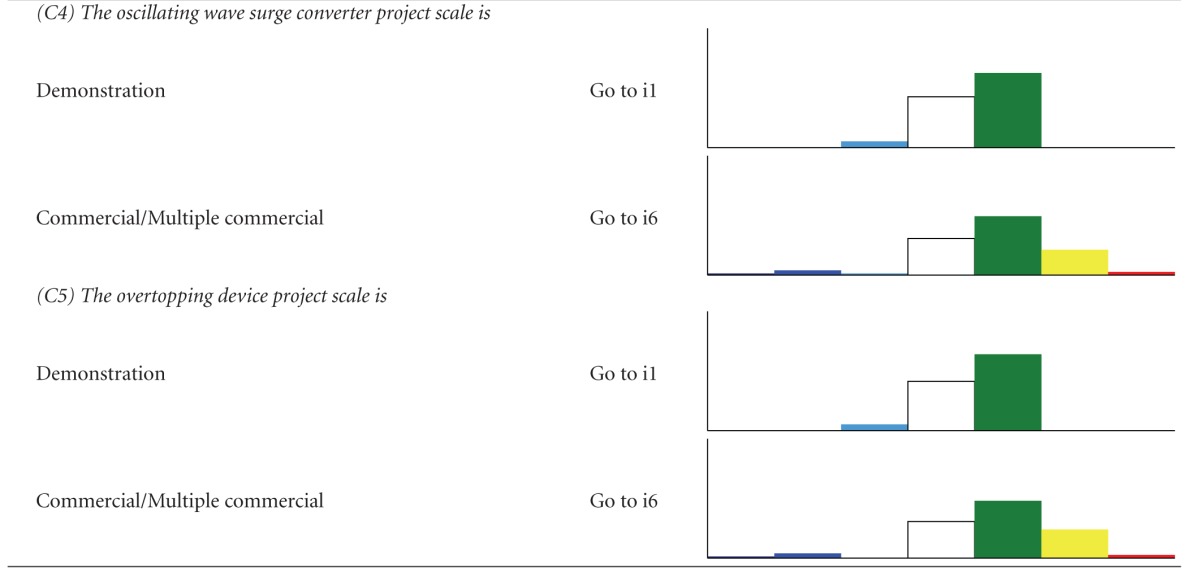

**Table 3:** A description of the potential impacts resulting from demonstration scale projects. See text, Section 3.3, for a narrative summary of this development scenario. Minor impacts are listed in the order they appear in the renewable energy impact matrix.

(i1) Demonstration scale projects		
Component (not ranked)	Minor impact	Certainty

Benthic habitat and resources	(i) Disturbance from installation/removal of device (including turbidity)	(i) High
	(ii) Disturbance from installation or removal of power cable (including trenching)	(ii) High
	(iii) Scour around structures	(iii) High
	(iv) Smothering by excavated sediments	(iv) High
	(v) Reef effects	(v) High
	(vi) Diffusion/flaking of marine coating	(vi) High
	(vii) Chemicals discharged during installation or removal	(vii) High
	(viii) Resuspension of pollutants in sediments	(viii) High

Fish species and fishing activity	(i) Disturbance from installation or removal of device	(i) High
	(ii) Disturbance from installation or removal or power cable	(ii) High
	(iii) Reef effects	(iii) High
	(iv) Loss of access to grounds during construction	(iv) High
	(v) Loss of access to grounds during operation	(v) High

Avian species	(i) Displacement or attraction to structure above surface of the water (wind turbines)	(i) High
	(ii) Displacement or attraction to structure below the surface of the water	(ii) High
	(iii) Disturbance from installation of device or transmission cable	(iii) High
	(iv) Collision with rotating turbine blades	(iv) High

**Table 4:** A description of the potential impacts resulting from wind turbine projects involving pile driving. See text, Section 3.3, for a narrative summary of this development scenario. Ecosystem components are ranked by their proportion of the major impacts. Moderate impacts are listed in the order they appear in the renewable energy impact matrix.

(i2) Wind turbines involving pile driving		
Priority	Major impacts	Certainty

(1) Fish species and fishing activity	Loss of access to grounds during construction and operation (mobile gear)	High
(2) Avian species*	Displacement or attraction to structure above water surface	High
(3) Benthic habitat and resources	Scour and/or deposition	High
(4) Marine mammals and sea turtles*	Noise from pile driving	Medium

Component	Moderate impacts	Certainty

Benthic habitat and resources	(i) Resuspension of pollutants in sediments	(i) High
	(ii) Chemical spills, discharge	(ii) Medium
	(iii) Disturbance from installation of cable	(iii) Medium
	(iv) Changes to current/wave regime	(iv) Medium

Fish species and fishing activity	(i) Chemical spills	(i) Medium
	(ii) Operational noise	(ii) Medium
	(iii) Noise from preconstruction seismic surveys	(iii) Medium
	(iv) Noise from pile driving	(iv) Medium
	(v) Noise from pile cutting during device removal	(v) Medium
	(vi) EMF	(vi) Low
	(vii) Habitat/community composition alteration	(vii) Medium
	(viii) Decreased catchability during construction and operation	(viii) Medium
	(ix) Loss of access to grounds during construction and operation (fixed gear and recreational)	(ix) High
	(x) Changes in species distribution	(x) Low
	(xi) Damaged/lost gear	(xi) High

Avian species	(i) Displacement or attraction to structure below water surface	(i) Medium
	(ii) Collision with rotating turbine blades	(ii) High
	(iii) Pressure gradients around rotor	(iii) Medium
	(iv) Leakage of lubricants/fluids, release of maintenance chemicals	(iv) Medium
	(v) Large chemical spills	(v) High

Marine mammals and sea turtles	(i) Entanglement with mooring lines or cables	(i) Medium
	(ii) Strikes with installation or support vessels	(ii) High
	(iii) Operational noise	(iii) Medium
	(iv) Noise from pile cutting during device removal	(iv) High

*Denotes that higher priority may be given to this component due to national/regional/local regulatory objectives and obligation.

**Table 5:** A description of the potential impacts resulting from wind turbine projects involving no pile driving. See text, Section 3.3, for a narrative summary of this development scenario. Ecosystem components are ranked by their proportion of the major impacts. Moderate impacts are listed in the order they appear in the renewable energy impact matrix.

(i3) Wind turbines involving no pile driving		
Priority	Major impacts	Certainty

(1) Fish species and fishing activity	Loss of access to grounds during construction and operation (mobile gear)	High
(2) Avian species*	Displacement or attraction to structure above water surface	High
(3) Benthic habitat and resources	Scour and/or deposition	High

Component	Moderate impacts	Certainty

Benthic habitat and resources	(i) Resuspension of pollutants in sediments	(i) High
	(ii) Disturbance from installation/removal of device (turbidity)	(ii) Medium
	(iii) Chemical spills, discharge	(iii) Medium
	(iv) Disturbance from installation of cable	(iv) Medium
	(v) Changes to current/wave regime	(v) Medium

Fish species and fishing activity	(i) Chemical spills	(i) Medium
	(ii) Operational noise	(ii) Medium
	(iii) Noise from pre-construction seismic surveys	(iii) Medium
	(iv) Noise from pile cutting during device removal	(iv) Medium
	(v) EMF	(v) Low
	(vi) Habitat/community composition alteration	(vi) Medium
	(vii) Decreased catchability during construction and operation	(vii) Medium
	(viii) Loss of access to grounds during construction and operation (fixed gear and recreational)	(viii) High
	(ix) Changes in species distribution	(ix) Low
	(x) Damaged/lost gear	(x) High

Avian species	(i) Displacement or attraction to structure below water surface	(i) Medium
	(ii) Collision with rotating turbine blades	(ii) High
	(iii) Pressure gradients around rotor	(iii) Medium
	(iv) Leakage of lubricants/fluids, release of maintenance chemicals	(iv) Medium
	(v) Large chemical spills	(v) High

Marine mammals and sea turtles	(i) Entanglement with mooring lines or cables	(i) Medium
	(ii) Strikes with installation or support vessels	(ii) High
	(iii) Operational noise	(iii) Medium

*Denotes that higher priority may be given to this component due to national/regional/local regulatory objectives and obligation.

**Table 6:** A description of the potential impacts resulting from bottom-mounted tidal turbine projects. See text, Section 3.3, for a narrative summary of this development scenario. Ecosystem components are ranked by their proportion of the major impacts. Moderate impacts are listed in the order they appear in the renewable energy impact matrix.

(i4) Bottom-mounted tidal turbine projects		
Priority	Major impacts	Certainty

(1) Benthic habitat and resources	(i) Changes in hydrodynamics (ii) Scour and/or deposition	(i) Medium (ii) High
(2) Fish species and fishing activity*	Loss of access to grounds during construction and operation (mobile gear)	High
(3) Marine mammals and sea turtles*	Noise from pile driving	Medium

Component	Moderate impacts	Certainty

Benthic habitat and resources	(i) Resuspension of pollutants in sediments	(i) Low
	(ii) Disturbance from installation/removal of device (turbidity)	(ii) High
	(iii) Chemical spills, discharge	(iii) Medium
	(iv) Disturbance from installation of cable	(iv) Medium
	(v) Changes to current/wave regime	(v) Medium

Fish species and fishing activity	(i) Collision/blade strike	(i) Medium
	(ii) Pressure gradients around rotor	(ii) Medium
	(iii) Chemical spills	(iii) Medium
	(iv) Operational noise	(iv) Medium
	(v) Noise from pre-construction seismic surveys	(v) Medium
	(vi) Noise from pile driving	(vi) Medium
	(vii) Noise from pile cutting during device removal	(vii) Medium
	(viii) EMF	(viii) Low
	(ix) Habitat/community composition alteration	(ix) Medium
	(x) Decreased catchability during construction and operation	(x) Medium
	(xi) Loss of access to grounds during construction and operation (fixed gear and recreational)	(xi) High
	(xii) Changes in species distribution	(xii) Low
	(xiii) Damaged/lost gear	(xiii) High

Avian species	(i) Collision with rotating turbine blades	(i) Medium
	(ii) Pressure gradients around rotor	(ii) Medium
	(iii) Leakage of lubricants/fluids, release of maintenance chemicals	(iii) Medium
	(iv) Large chemical spills	(iv) High

Marine mammals and sea turtles	(i) Entanglement with mooring lines or cables	(i) Medium
	(ii) Strikes with installation or support vessels	(ii) High
	(iii) Operational noise	(iii) Medium
	(iv) Noise from pile cutting during device removal	(iv) High

*Denotes that higher priority may be given to this component due to national/regional/local regulatory objectives and obligation.

**Table 7:** A description of the potential impacts resulting from floating mooring tidal turbine projects. See text, Section 3.3, for a narrative summary of this development scenario. Ecosystem components are ranked by their proportion of the major impacts. Moderate impacts are listed in the order they appear in the renewable energy impact matrix.

(i5) Floating mooring tidal turbine projects		
Priority	Major impacts	Certainty

(1) Benthic habitat and resources	(i) Changes in hydrodynamics (ii) Scour and/or deposition	(i) Medium (ii) High
(2) Fish species and fishing activity*	Loss of access to grounds during construction and operation (mobile gear)	High

Component	Moderate impacts	Certainty

Benthic habitat and resources	(i) Resuspension of pollutants in sediments	(i) Low
	(ii) Chemical spills, discharge	(ii) Medium
	(iii) Disturbance from installation of cable	(iii) Medium
	(iv) Changes to current/wave regime	(iv) Medium

Fish species and fishing activity	(i) Collision/blade strike	(i) Medium
	(ii) Pressure gradients around rotor	(ii) Medium
	(iii) Chemical spills	(iii) Medium
	(iv) Operational noise	(iv) Medium
	(v) Noise from pre-construction seismic surveys	(v) Medium
	(vi) EMF	(vi) Low
	(vii) Habitat/community composition alteration	(vii) Medium
	(viii) Loss of access to grounds during construction and operation (fixed gear and recreational)	(viii) High
	(ix) Changes in species distribution	(ix) Low
	(x) Damaged/lost gear	(x) High

Avian species	(i) Collision with rotating turbine blades	(i) Medium
	(ii) Pressure gradients around rotor	(ii) Medium
	(iii) Leakage of lubricants/fluids, release of maintenance chemicals	(iii) Medium
	(iv) Large chemical spills	(iv) High

Marine mammals and sea turtles	(i) Entanglement with mooring lines or cables	(i) Medium
	(ii) Strikes with installation or support vessels	(ii) High
	(iii) Operational noise	(iii) Medium

*Denotes that higher priority may be given to this component due to national/regional/local regulatory objectives and obligation.

**Table 8:** A description of the potential impacts resulting from projects harnessing wave energy. See text, Section 3.3, for a narrative summary of this development scenario. Ecosystem components are ranked by their proportion of the major impacts. Moderate impacts are listed in the order they appear in the renewable energy impact matrix.

(i6) Wave energy projects		
Priority	Major impacts	Certainty

(1) Benthic habitat and resources	(i) Changes in hydrodynamics	(i) Medium
	(ii) Scour and/or deposition	(ii) High
(2) Fish species and fishing activity*	Loss of access to grounds during construction and operation (mobile gear)	High

Component	Moderate impacts	Certainty

Benthic habitat and resources	(i) Resuspension of pollutants in sediments	(i) Low
	(ii) Chemical spills, discharge	(ii) Medium
	(iii) Disturbance from installation of cable	(iii) Medium
	(iv) Changes to current/wave regime	(iv) Medium

Fish species and fishing activity	(i) Chemical spills	(i) Medium
	(ii) Operational noise	(ii) Medium
	(iii) Noise from pre-construction seismic surveys	(iii) Medium
	(iv) EMF	(iv) Low
	(v) Habitat/community composition alteration	(v) Medium
	(vi) Decreased catchability during construction/operation	(vi) Medium
	(vii) Loss of access to grounds during construction and operation (fixed gear and recreational)	(vii) High
	(viii) Changes in species distribution	(viii) Medium
	(ix) Damaged/lost gear	(ix) High

Avian species	(i) Displacement/attraction to structure above water surface	(i) Medium
	(ii) Leakage of lubricants/fluids, release of maintenance chemicals	(ii) Medium
	(iii) Large chemical spills	(iii) High

Marine mammals and sea turtles	(i) Entanglement with mooring lines or cables	(i) Medium
	(ii) Strikes with installation or support vessels	(ii) High
	(iii) Operational noise	(iii) Medium

*Denotes that higher priority may be given to this component due to national/regional/local regulatory objectives and obligation.

**Table 9:** Component decision tree for impacts on benthic habitat and resources. The key is followed to obtain a recommended suite of monitoring protocols.

Determine which impacts to the benthic environment need to be monitored:	
*The energy resource is *	
Wind	Go to A
Waves	Go to B
Tidal	Go to C
*(A) The wind turbine foundation is *	
Monopile OR tripod OR lattice	Go to A1
Gravity	Go to A2
Floating mooring	Go to A3
* (A1) The stage of the monopile, tripod, or lattice wind turbine project is *	
Construction	Z1, Z2
Operation	Z1, Z2, Z3
Decommissioning	Z1, Z2
* (A2) The stage of the gravity wind turbine project is *	
Construction	Z1, Z2, Z3
Operation	Z1, Z2, Z3
Decommissioning	Z1, Z2, Z3
* (A3) The stage of the floating mooring wind project is *	
Construction	Z1, Z2
Operation	Z1, Z2
Decommissioning	Z1, Z2
*(B) The tidal turbine type is *	
Open OR shrouded bottom mounted	Go to B1
Open OR shrouded floating mooring	Go to B2
* (B1) The stage of the bottom-mounted tidal turbine project is *	
Construction	Z1, Z2
Operation	Z1, Z2, Z3, Z4
Decommissioning	Z1, Z2
* (B2) The stage of the floating mooring tidal turbine project is *	
Construction	Z1, Z2
Operation	Z1, Z2, Z4
Decommissioning	Z1, Z2
*(C) The wave device type is *	
Point absorber OR wave attenuator OR oscillating water column	Go to C1
Oscillating wave surge converter	Go to C2
Overtopping	Go to C3
* (C1) The stage of the point absorber OR wave attenuator OR oscillating water column project is *	
Construction	Z1, Z2
Operation	Z2, Z4
Decommissioning	Z1, Z2
* (C2) The oscillating wave surge converter project scale is *	
Construction	Z1, Z2
Operation	Z1, Z2, Z3, Z4
Decommissioning	Z1, Z2
* (C3) The overtopping device project scale is *	
Construction	Z1, Z2
Operation	Z1, Z2, Z4
Decommissioning	Z1, Z2

Recommended protocols:

Z1: seabed scour and/or deposition.

Z2: changes in benthic community composition.

Z3: increase in hard bottom habitat.

Z4: changes in hydrodynamics.

**Table 10:** Component decision tree for impacts on fish species and fisheries. The key is followed to obtain a recommended suite of monitoring protocols.

Determine which impacts to fisheries resources and fishing activity need to be monitored:	
*The energy resource is *	
Wind	Go to A
Tidal	Go to B
Waves	Go to C
*(A) The wind turbine foundation is *	
Monopile OR tripod OR lattice OR gravity	Go to A1
Floating mooring	Go to A3
* (A1) The stage of the monopile, tripod, lattice, or gravity wind turbine project is *	
Construction	X1, X2, X5
Operation	X1, X2, X3, X5
Decommissioning	X1, X2
* (A2) The stage of the floating mooring wind project is *	
Construction	X1, X2, X5
Operation	X3, X4, X5
Decommissioning	X1, X2
*(B) The tidal turbine type is *	
Open OR shrouded bottom mounted	Go to B1
Open OR shrouded floating mooring	Go to B2
* (B1) The stage of the bottommounted tidal turbine project is *	
Construction	X1, X2, X5
Operation	X1, X2, X3, X4, X5
Decommissioning	X1, X2
* (B2) The stage of the floating mooring tidal turbine project is *	
Construction	X1, X2, X5
Operation	X1, X2 X3, X4, X5
Decommissioning	X1, X2
*(C) The wave device type is *	
Point absorber OR wave attenuator OR oscillating water column OR overtopping	Go to C1
Oscillating wave surge converter	Go to C2
* (C1) The stage of the point absorber OR wave attenuator OR oscillating water column OR overtopping project is *	
Construction	X1, X2, X5
Operation	X1, X2, X5
Decommissioning	X1, X2
* (C2) The oscillating wave surge converter project scale is *	
Construction	X1, X2, X5
Operation	X1, X2, X3, X5
Decommissioning	X1, X2

Recommended protocols:

X1: mesoscale changes to abundance and distribution (disturbance).

X1a: the species of concern are finfish.

X1b: the species of concern are crustaceans or rock fish.

X2: habitat alteration/community composition: microscale changes to abundance and distribution—finfish.

X3: reef effects.

X4: blade strikes.

X5: spatial use of fishing activity.

**Table 11:** Component decision tree for impacts on avian species. The key is followed to obtain a recommended suite of monitoring protocols.

Determine which impacts to avian species need to be monitored:	
*The energy resource is *	
Wind	Go to A
Waves	Go to B
Tidal	Go to C
*(A) The stage of the wind energy project is *	
Construction	Go to D
Operation	Go to D, Go to E, V11
Decommissioning	Go to D
*(B) The stage of the tidal project is *	
Construction	Go to D
Operation	Go to D, V12
Decommissioning	Go to D
*(C) The stage of the wave energy project is *	
Construction	Go to D
Operation	Go to D
Decommissioning	Go to D
* (D) The target species are *	
Easily disturbed, cryptic	V3, V4
Easily disturbed, noncryptic	V2, V3, V4
Not easily disturbed, cryptic	V1, V3, V4
Not easily disturbed, noncryptic	V1, V2, V3, V4
* (E) The target species are *	
Diurnal	V5, V6
Nocturnal	V5, V7

Recommended protocols:

V1: ship-based visual surveys.

V2: aerial surveys using human observers.

V3: aerial surveys using high-definition videography.

V4: aerial surveys using digital still photography.

V5: radar surveys.

V6: visual surveys.

V7: flight call surveys.

V11: remote detection system.

V12: sonar and video technology.

**Table 12:** Component decision tree for impacts on marine mammals. The key is followed to obtain a recommended suite of monitoring protocols.

Determine which impacts to marine mammals need to be monitored:	
*The energy resource is *	
Wind	Go to A
Tidal	Go to B
Waves	Go to C
*(A) The stage of the wind energy project is *	
Construction	W1, W2, W3, W4, W5
Operation	W1, W2, W3, W4, W5
Decommissioning	W1, W2, W3, W4, W5
*(B) The stage of the tidal energy project is *	
Construction	W1, W2, W3, W4, W5
Operation	W1, W2, W3, W4, W5
Decommissioning	W1, W2, W3, W4, W5
*(C) The stage of the wave energy project is *	
Construction	W1, W2, W3, W4, W5
Operation	W1, W2, W3, W4, W5
Decommissioning	W1, W2, W3, W4, W5

Recommended protocols:

W1: visual surveys.

W2: passive acoustic monitoring.

W3: marine mammal observers.

W4: stranding response networks.

W5: tagging.

W6/7: underwater photography.

W8: SCUBA surveys.

W9: ROV surveys.

**Table 13:** Component decision tree for impacts on sea turtles. The key is followed to obtain a recommended suite of monitoring protocols.

Determine which impacts to sea turtles need to be monitored:	
*The energy resource is *	
Wind	Go to A
Tidal	Go to B
Waves	Go to C
*(A) The wind turbine foundation is *	
Monopile OR tripod OR floating mooring	Go to A1
Lattice OR gravity	Go to A2
* (A1) The stage of the monopile or tripod or floating mooring wind turbine project is *	
Construction	W1, W3, W4, W5, W8
Operation	W1, W3, W4, W5
Decommissioning	W1, W3, W4, W5, W8
* (A2) The stage of the lattice structure or gravity foundation wind project is *	
Construction	W1, W3, W4, W5, W8
Operation	W1, W3, W4, W5
Decommissioning	W1, W3, W4, W5, W6, W7, W8
*(B) The tidal turbine type is *	
Open OR shrouded bottom-mounted	Go to B1
Open OR shrouded floating mooring	Go to B2
* (B1) The stage of the bottommounted tidal turbine project is *	
Construction	W1, W3, W4, W5, W8
Operation	W1, W3, W4, W5
Decommissioning	W1, W3, W4, W5, W6, W7, W8
* (B2) The stage of the floating mooring tidal turbine project is *	
Construction	W1, W3, W4, W5, W8
Operation	W1, W3, W4, W5
Decommissioning	W1, W3 W4, W5, W8
*(C) The stage of the wave energy device is *	
Construction	W1, W3, W4, W5, W8
Operation	W1, W3, W4, W5
Decommissioning	W1, W3, W4, W5, W8

Recommended protocols:

W1: visual surveys.

W2: passive acoustic monitoring.

W3: marine mammal observers.

W4: stranding response networks.

W5: tagging.

W6: underwater photography.

W7: SCUBA surveys.

W8: ROV surveys.

**Table 14:** Monitoring protocols relevant to addressing the potential impacts identified from the Impact decision tree and component decision trees for the case study of a scale 2 (~200 devices) jacketed wind turbine farm involving pile driving. Protocols in bold face represent those that uniquely address the Impact scenario and local concerns and should thus be prioritized for implementation.

benthic habitat and resources:	
**(Z1) Seabed scour and/or deposition**	
(Z2) Changes in benthic community composition	
(Z3) Increase in hard bottom habitat	
Fish and fisheries resources	
(X1a) Trawl surveys	
** (X1b) Ventless trap surveys**	
(X2) Habitat alteration/community composition: micro-scale changes to abundance and distribution—finfish	
(X3) Reef effects	
**(X5) Spatial use of fishing activity **	
Avian species	
**(V3) Aerial surveys using high-definition videography**	
**(V4) Aerial surveys using digital still photography**	
(V5) Radar surveys	
(V6) Visual surveys of flight ecology	
(V11) Remote detection system	
Marine mammals/sea turtles	
**(W1) Visual surveys**	
**(W2) Passive acoustic monitoring**	
**(W3) Marine mammal observers**	
(W4) Stranding response networks	
(W5) Tagging	
(W8) ROV surveys	

**Table 15:** An example monitoring protocol from the benthic habitat and resources category for seabed scour and/or deposition at a commercial-scale installation.

	High cost	Low cost
Indicator(s) of the impact	Scour: increase in median grain size, decrease in organic content, decrease in seabed volume	
	Deposition: decrease in median grain size, increase in organic content, increase in seabed volume	

Methodology or technique to collect data	Particle size analysis, multibeam/interferometric bathymetry	

Description of methodology or technique(s) for collecting data	Seasonal surveys, 5 years	Annual surveys, 3 years
	Grain size: *5-sample transect at 3 devices out to 200 m	Grain size: *3-sample transect at 3 devices out to 200 m
	Bathymetry: overlapping transects for 100% coverage (at least 0.5 m pixels) 1 km radius at 3 devices	Bathymetry: overlapping transects for 100% coverage (at least 0.5 m pixels) 500 m radius 3 devices

Methodology for analyzing data	ANOVA on median grain size, volume change estimate using mosaicked bathymetry models	

Frequency and duration	1 preconstruction survey, seasonal operation, 1 postconstruction survey	1 preconstruction survey, annual operation, 1 postconstruction survey

Spatial scale	200 m–1 km radius around 3 devices	500 m radius around 3 devices

How well does this methodology account for environmental variability?	Seasonal and interannual variability	Interannual variability

Cost	$25 k/year, 5 years	$15 k/year, 3 years

Other considerations (e.g., advantages or disadvantages)	Can be combined with benthic community composition monitoring protocol	

Type of data output required: time series values for median grain size and standard deviations, time series on volume at each turbine and standard deviation.
