# Supplementary material for: An Adaptive Framework for Selecting Environmental Monitoring Protocols to Support Ocean Renewable Energy Development
Source: ScientificWorldJournal. 2012 Dec 23;2012:450685. doi: 10.1100/2012/450685 (PMC3540755; doi:10.1100/2012/450685)
Supplement: Supplementary file 1 — The online supplementary material consists of a list of works cited from a literature review of the environmental impacts of renewable energy developments (Appendix A) and a series of tables that summarize the major impacts on multiple ecosystem components at two scales (Appendix B). We grouped impacts by ecosystem component: benthic habitat and resources, fish and fisheries resources, avian species, marine mammals and sea turtles. We further divided impacts by scale: “scale 1” represents demonstration-scale developments consisting of fewer than three devices and “scale 2” represents commercial-scale developments consisting of 100 or more devices. Finally, we differentiate impacts based on renewable energy device and foundation type. This impact assessment provided the foundation for developing decision support tools based on affected ecosystem component, scale, and device type. [file 450685.f1.pdf]

Appendix A. Works cited in offshore renewable energy impacts literature review.

ABPmer Ltd, et al., 2010. A Further Review of Sediment Monitoring Data. Commissioned by COWRIE Ltd (project reference ScourSed-09), 115 pp.

Addis, P., Cau, A., Massutí, E., Merella, P., Sinopoli, M., Andaloro, F., 2006. Spatial and temporal changes in the assemblage structure of fish associated to fish aggregation devices in the Western Mediterranean. Aquatic Living Resources 19: 149-160.

Allen, B.M., Angliss, R.P., 2010. Alaska Marine Mammal Stock Assessments, 2009. NOAA Technical Memorandum NMFS-AFSC-206.National Marine Fisheries Service, Seattle, WA.

AMEC Wind Energy. 2002. Lynn Offshore Environmental Impact Assessment.

Andersen, B., Jakobsen, J., 1983. Noise Emission from Wind Turbine Generators: A Measurement Method. Danish Acoustical Institute Technical Report No. 109, Lyngby, Denmark: Danish Academy of Technical Sciences.

Anderson, J.T., Van Holliday, D., Kloser, R., Reid, D., Simard, Y., 2008. Acoustic seabed classification: Current practice and future directions. ICES Journal of Marine Science 65: 1004-1011.

Anderson, R.L., Morrison, M., Sinclair, K., Strickland, M.D., 1999. Studying wind energy–bird interactions: a guidance document. Prepared for avian subcommittee and National Wind Coordinating Committee., http://www.nationalwind.org/publications/wildlife/avian99/Avian_booklet.pdf.

Arnett, E.B, Brown, W.K., Erickson, W.P., Fiedler, J.K., Hamilton, B.L., Henry, T.H., Jain, A., Johnson, G.D., Kerns, J., Koford, R.K., Nicholson, C.P., O'Connell, T.J., Piorkowski, M.D., Tankersley, Jr., R.D., 2008. Patterns of bat fatalities at wind energy facilities in North America. Journal of Wildlife Management 72: 61-78.

Bachmann, S., Zrnic, D.S., 2007. Spectral density of polarimetric variables separating biological scatterers in the VAD display. Journal of Atmospheric and Oceanic Technology 24: 1186-1198.

Baerwald, E. F., D'Amours, G.H., Klug, B.J., Barclay, R.M.R., 2008. Barotrauma is a significant cause of bat fatalities at wind turbines. Current Biology 18: 695-696.

Bailey, H., Seniro, B., Simmons, D., Rusin, J., Picken, G., Thompson, P.M., 2010. Assessing underwater noise levels during pile-driving at an offshore windfarm and its potential effect on marine mammals. Marine Pollution Bulletin 60: 888-897.

Baird, P.H., 1990. Concentrations of seabirds at oil-drilling rigs. Condor 92: 768-

771.
Balmori, A., 2005 Possible effects of electromagnetic field from phone masts on a population of white stork (Ciconia ciconia). Electromagnetic Biology and Medicine 24: 109-119.
Balmori, A., 2009. Electromagnetic pollution from phone masts: Effects on wildlife. Pathophysiology doi:10.1016/j.pathophys.2009.01.007.
Barclay, R.M.R., Baerwald, E.F., Gruver, J.C., 2007. Variation in bat and bird fatalities at wind energy facilities: assessing the effects of rotor size and tower height. Canadian Journal of Zoology 85: 381-287.
Barlow, J., Ferguson, M.C., Becker, E.A., Redfern, J.V., Forney, K.A., Vilchis, I.L., Fiedler, P.C., Gerrodette, T., Balance, L.T., 2009. Predictive Modeling of Cetacean Densities in the Eastern Pacific Ocean. NOAA Tech Memo NMFS-SWFSC-444. National Marine Fisheries Service, Southwest Fisheries Science Center, La Jolla, CA.
Barrios, L., Rodríquez, A., 2004. Behavioral and environmental correlates of soaring-bird mortality at on-shore wind turbines. Journal of Applied Ecology 41: 72-81.
Barrow Offshore Wind Ltd. 2009. Barrow Offshore Wind Farm Post-Construction Monitoring Report. DONG Energy, Copenhagen, Denmark.
Becker E.A., Forney, K.A., Ferguson, M.C., Foley, D.G., Smith, R.C., Barlow, J., Redfern, J.V., 2010. Comparing California Current cetacean-habitat models developed using in situ and remotely sensed sea surface temperature data. Marine Ecology Progress Series 413: 163–183.
Bergman, M., Duineveld, G., Hof, P.V., 2008. Interim Report: Benthos Recruitment T1. Report OWEZ_R_262_T1_20080222 draft, 49 pp.
Birklund, J., Petersen, A.H., 2004. Development of the fouling community on turbine foundations and scour protections in Nysted Offshore Wind Farm, 2003. Energi E2 A/S.
Blehert, D.S., Hicks, A.C., Behr, M., Meteyer, M.U., Berlowski-Zier, B.M., Buckles, E.L., Coleman, J.T.H., Darling, S.R., Gargas, A., Niver, R., Okoniewski, J.C., Rudd, R.J., Stone, W.B., 2009. Bat White-nose Syndrome: An emerging fungal pathogen? Science 323: 227.
Blew, J., Hoffmann, M., Nehls, G., Hennig, V., 2008. Investigations of the bird collision risk and the responses of harbor porpoises in the offshore wind farms Horns Rev, North Sea, and Nysted. Report funded by German Federal Ministry for the Environment, Nature Conservation and Nuclear Safety.
Bloor, P., Wratten, A., 2006. Aerial surveys of waterbirds in strategic wind farm areas: 2004/05 Final Report. Department of Trade and Industry DTI/Pub 8298, UK.

Boehlert, G.W., Gill, A.B., 2010. Environmental and ecological effect of ocean renewable energy development - a current synthesis. Oceanography 23(2): 64-77.

Boehlert, G.W., McMurray, G.R., Tortorici, C.E., (Eds.), 2008. Ecological Effects of Wave Energy Development in the Pacific Northwest. U.S. Department of Commerce, NOAA Technical Memorandum NMFS-F/SPO-92.

Borberg, J.M., Balance, L.T., Pittman, R.L., Ainley, D.G., 2005. A test for bias attributable to seabird avoidance of ships during surveys conducted in the tropical Pacific. Marine Ornithology 33: 173-179.

Borja, A., Josefson, A.B., Miles, A., Muxika, I., Olsgard, F., Phillips, G., Rodríguez, J.G., Rygg, B., 2007. An approach to the intercalibration of benthic ecological status assessment in the North Atlantic ecoregion, according to the European Water Framework Directive. Marine Pollution Bulletin 55: 42-52.

Bouma, S., Lengkeek, W., 2009. Development of underwater flora- and fauna communities on hard substrates of the offshore wind farm Egmond aan Zee (OWEZ). Report nr 08-220. Bureau Waardenburg bv, Nordzeewind, 46 pp.

Brandt, M.J., Diederichs, A., Betke, K., Nehls, G., 2011. Responses of harbour porpoises to pile driving at the Horns Rev II offshore wind farm in the Danish North Sea. Marine Ecology Progress Series 421: 205-216.

Breton, S.P., Moe, G., 2009. Status, plans, and technologies for offshore wind turbines in Europe and North America. Renewable Energy, 34:646-654.

Briggs, K.T., Tyler, W.B., Lewis, D.B., 1985. Comparison of ship and aerial surveys of birds at sea. Journal of Wildlife Management 49: 405-411.

Brown, C.J., Collier, J.S., 2008. Mapping benthic habitat in regions of gradational substrata: An automated approach utilising geophysical, geological, and biological relationships. Estuarine Coastal and Shelf Science 78: 203-214.

Buckland, S.T., 2001. Introduction to distance sampling: estimating abundance of biological populations. Oxford University Press.

Buckland, S.T., 2004. Advanced Distance sampling. Oxford University Press.

BSH (Bundesamt für Seeschifffahrt und Hydrographie), 2007. Standard Investigation of the Impacts of Offshore Wind Turbines on the Marine Environment (StUK 3). BSH-Nr. 7003, Bundesamt für Seeschifffahrt und Hydrographie, Hamburg and Rostock, Germany. Available online from: http://www.bsh.de/en/Products/Books/Standard/7003eng.pdf

Burger, A.E. Fry, D.M., 1993. Effects of oil pollution on seabirds in the northeast Pacific. In: Vermeer, K., Briggs, K.T., Morgan, K.H., Siegel-Causey, D. (Eds.), The Status, Ecology and Conservation of Marine Birds of the North Pacific, Canadian Wildlife Service, Ottawa , pp. 254–263.

Burger, J., Gochfeld, M., 2002. Effects of Chemicals and Pollution on Seabirds. In: Schreiber, E.A., Burger, J. (Eds.), Biology of Marine Birds. CRC Press, Boca Raton, Florida, pp. 485-526.
Burger, J., Gordon, C., Lawrence, J., Newman, J., Forcey, G., Vlietstra, L., 2011. Risk evaluation for federally listed (roseate tern, piping plover) or candidate (red knot) bird species in offshore waters: A first step for managing the potential impacts of wind facility development on the Atlantic Outer Continental Shelf. Renewable Energy 36: 338-351.
Burt, L., Rexstad, E., Buckland, S., 2009. Comparison of visual and digital aerial survey results of avian abundance for Round 3, Norfolk Region. COWRIE Ltd., United Kingdom.
Burt, L., Rexstad, E., Buckland, S., 2010. Comparison of design- and model-based estimates of seabird abundance derived from visual, digital still transects and digital still transects and digital video aerial surveys in Carmarthen Bay. CREEEM for Cowrie Ltd.
Burton, N.H.K., Thaxter, C.B., Cook, A.S.C.P., Austin, G.E., Wright, L.J., Clark, N.A., 2010. Evaluating the potential impacts of tidal power schemes on estuarine waterbirds. BOU Proceeding – Climate change and birds. Accessed at www.bouproc.net.
Caltrans, 2001. Fisheries Impact Assessment: San Francisco – Oakland Bay Bridge East Span Seismic Safety Project, Pile Installation Demonstration Project. Caltrans Contract 04A0148.
Camphuysen, C.J., Fox, A.D., Leopold, M.F., Petersen, I.K., 2004. Towards standardised seabirds at sea census techniques in connection with environmental impact assessments for offshore winds in the U.K. A comparison of ship and aerial sampling methods for marine birds, and their applicability to offshore wind farm assessments. Koninklijk Nederlands Instituut Onderzoek der Zee Report commissioned by COWRIE.
Carey, J.M., Keough, M.J., 2002. The variability of estimates of variance, and its effect on power analysis in monitoring design. Environmental Monitoring and Assessment 74: 225-41.
Carretta, J.V., Forney, K.A., Lowry, M.S., Barlow, J., Baker, J., Johnston, D., Hanson, B., Brownell Jr., R.M., Robbins, J., Mattila, D.K., Ralls, K., Muto, M.M., Lynch, D., Carswell, L., 2010. U.S. Pacific Marine Mammal Stock Assessments: 2009. NOAA Technical Memorandum NMFS-SWFSC-453. National Marine Fisheries Service, La Jolla, CA.
Carstensen, J., Henriksen, O.D., Teilmann, J., 2006. Impacts of offshore wind farm

construction on harbour porpoises: acoustic monitoring of echolocation activity using porpoise detectors (T-PODs). Marine Ecology Progress Series 321: 295-308.
CEFAS, 2010. Strategic Review of Offshore Wind Farm Monitoring Data Associated with FEPA License Conditions, Report #ME1117.
CETAP (Cetacean and Turtle Assessment Program, University of Rhode Island), 1982. A Characterization of Marine Mammals and Turtles in the Mid- and North Atlantic Areas of the U. S. Outer Continental Shelf, Final Report. Contract AA551-CT8-48.Bureau of Land Management, Washington, DC.
Chamberlain, D.E., Rehfisch, M.R., Fox, A.D., Desholm, M., Anthony, S.J., 2006. The effect of avoidance rates on bird mortality predictions made by wind turbine collision risk models. Ibis 148: 198-202.
Champ, M.A., 2003. Economic and environmental impacts on ports and harbors from the convention to ban harmful marine anti-fouling systems. Marine Pollution Bulletin 46: 935–940.
Christenesen, T.K., Hounisen, J.P., Clausager, I., Petersen, I.K., 2004. Visual and radar observations of birds in relation to collision risk at the Horns Rev. offshore wind farm. Annual status report 2003. Report commissioned by Elsam Engineering A/S 2003. NERI Report. Ronde, Denmark: National Environmental Research Institute.
Christensen, T.K., Clausager, I., Petersen, I.K., 2003. Base-line investigations of birds in relation to an offshore wind farm at Horns Rev. NERI Report, Denmark.
Christensen, T.K., Petersen, I.K., Fox, A.D., 2006. Effects on birds of the Horns Rev 2 offshore wind farm: Environmental Impact Assessment. NERI Report.
Clark, N.A., 2006. Tidal barrages and birds. Ibis 148: 152-157.
Clarke, K.R., Gorley, R.N., 2006. Primer-E, version 6. Plymouth, UK.
Clausen, K. T., Wahlberg, M., Beedholm, K., Deruiter, S., Madsen, P.T., 2010. Click communication in harbour porpoises Phocoena phocoena. Bioacoustics 20: 1-28.
Commonwealth of Massachusetts, 2009. Massachusetts Ocean Plan - Volume 2 - Baseline Assessment. State of Massachusetts Office for Energy and Environmental Affairs, 253 pp.
COWRIE/CMACS, 2003. A baseline assessment of electromagnetic fields generated by offshore windfarm cables. Center for Marine and Coastal Studies, University of Liverpool, UK.
Cranswick, P., Hall, C., Smith, L., 2003. Aerial surveys of birds in proposed strategic areas for offshore windfarm development, Round 2: preliminary report, winter 2003/03. Wildfowl and Wetlands Trust Research Report, Slimbridge, Gloucestershire, UK.

Crawford R.L., Engstrom, R.T., 2001. Characteristics of avian mortality at a north Florida television tower: a 29-year study. Journal of Field Ornithology 72: 380–388.

Cryan, P.M., Brown, A.C., 2007. Migration of bats past a remote island offers clues toward the problem of bat fatalities at wind turbines. Biological Conservation 139 (1-2): 1-11.

Daan, R., Mulder, M., Bergman, M.J.N., 2009. Impact of windfarm OWEZ on the local macrobenthos community. Report OWEZ_R_261_T1_20091216, 77 pp.

David, J.A., 2006. Likely sensitivity of bottlenose dolphins to pile-driving noise. Water and Environment Journal 20: 48-54.

de Lestang, S., Caputi, N., How, J., Melville-Smith, R., Thomson, A., Stephenson, P., 2011. Draft Stock Assessment for the West Coast Rock Lobster Fishery. Government of Western Australia, Department of Fisheries.

DEFRA, 2005. Nature Conservation Guidance on Offshore Windfarm Development - A guidance note on the implications of the EC Wild Birds and Habitats Directives for developers undertaking offshore windfarm developments. Available at: http://webarchive.nationalarchives.gov.uk/20080915101357/http://www.defra.gov.uk/wildlife-countryside/ewd/windfarms/index.htm

Degraer, S., Brabant, R., Rumes, B. (Eds.), 2010. Offshore wind farms in the Belgian part of the North Sea: Early environmental impact assessment and spatio-temporal variability. Royal Belgian Institute of Natural Sciences, Management Unit of the North Sea Mathematical Models. Marine ecosystem management unit. 184 pp. + annexes.

Degraer, Steven, Brabant, R. (Eds.), 2009. Offshore wind farms in the Belgian part of the North Sea: State of the art after two years of environmental monitoring; Chapter 12: Recommendations for a future monitoring of wind farms in Belgium's marine waters. Royal Belgian Institute for Natural Sciences, Management Unit of the North Sea Mathematical Models. Marine ecosystem management unit. pp. 275-279.

Dempster, T., Taquet, M., 2004. Fish aggregation device (FAD) research: Gaps in current knowledge and future directions for ecological studies. Reviews in Fish Biology and Fisheries 14: 21–42.

Derweduwen, J., Vandendriessche, S., Hostens, K., 2010. Monitoring of the effects of the Thorntonbank and Bligh Bank wind farms on the epifauna and demersal fish fauna of soft-bottom sediments: Thorntonbank: status during construction (T2), Bligh Bank: status during construction (T1). In: Degraer, S., Brabant, R. & Rumes, B. (Eds.) 2010. Offshore wind farms in

the Belgian part of the North Sea: Early environmental impact assessment and spatio-temporal variability. Royal Belgian Institute of Natural Sciences, Management Unit of the North Sea Mathematical Models. Marine ecosystem management unit. pp. 105-131.

Desholm, M.D., 2005. TADS investigations of avian collision risk at Nysted offshore wind farm. NERI Report, 2005.

Desholm, M.D., 2005a. Preliminary investigations of bird-turbine collisions at Nysted offshore wind farm and final quality control of Thermal Animal Detection System (TADS); autumn 2003 and spring 2004. NERI Report. Ronde, Denmark: National Environmental Research Institute.

Desholm, M.D., 2005b. TADS investigations of avian collision risk at Nysted offshore wind farm, autumn 2004. NERI Report. Ronde, Denmark: National Environmental Research Institute.

Desholm, M.D., 2009. Avian sensitivity to mortality: prioritizing migratory bird species for assessment at proposed wind farms. Journal of Environmental Management 90: 2672-2679.

Desholm, M.D., Kahlert, J., 2005. Avian collision risk at an offshore wind farm. Biology Letters 1: 296–298.

Desholm, M.D., Fox, A.D., Beasley, P.D.L., 2004. Best practice guidance for the use of remote techniques for observing bird behaviour in relation to offshore wind farms, COWRIE-REMOTE-05-2004.

Desholm, M.D., Fox, A.D., Beasley, P.D.L., Kahlert, J., 2006. Remote techniques for counting and estimating the number of bird-wind turbine collisions at sea: a review. Ibis 148: 76-89.

Dhanju, A., Firestone, J., 2008. A framework for regulation of offshore wind power in Delaware state waters. Unpublished report, University of Delaware College of Marine and Earth Studies.

Diaz, R.J., Solan, M., Valente, R.M., 2004. A review of approaches for classifying benthic habitats and evaluating habitat quality. Journal of Environmental Management 73: 165-181.

Diederichs, A., Grünkhorn, T., Nehls, G., 2008. Offshore wind farms—disturbance or attraction for harbour porpoises? First results of T-POD investigations in Horns Rev and Nysted, In: Evans, P.G.H. (Ed.), Proceedings of the ASCOBANS/ECS Workshop; Offshore Wind Farms and Marine Mammals: Impacts and Methodologies for Assessing Impacts. ECS Special Publication Series No. 49, European Cetacean Society, Caeernarfon, Gwynedd, UK. pp. 42-49.

Dolman, S.J., Green, M., Simmonds, M.P., 2007. Marine Renewable Energy and Cetaceans. SC/59/E10 Submitted to the Scientific Committee of the International Whaling Commission.

DoN (U.S. Department of the Navy), 2008. Final Atlantic Fleet Active Sonar Training Environmental Impact Statement/Overseas Environmental Impact Statement. Department of the Navy, U.S. Naval Facilities Engineering Command, Atlantic, Norfolk, VA. 1754 pp.

DoN (U.S. Department of the Navy), 2009a. Report on the OPNAV N45 Marine Mammal Monitoring Workship; R. David Thomas Executive Conference Center, Duke University, Durham, NC; February 19-20, 2009. U.S. Navy, Chief of Naval Operations Environmental Readiness Division, Washington, DC. 14 pp.

DoN (U.S. Department of the Navy), 2009b. United States Navy Integrated Comprehensive Monitoring Program. U.S. Navy, Chief of Naval Operations Environmental Readiness Division, Washington, DC. 50 pp.

DONG Energy, 2006. Danish Offshore Wind - Key Environmental Issues. Chapter 5: Fish. DONG Energy, Vattenfall, the Danish Energy Authority and The Danish Forest and Nature Agency, Denmark.

Dooling, R., 2002. Avian hearing and avoidance of wind turbines. Technical Report. NREL/TP-500-30844, Golden, Colorado.

Drewitt, A.L, Langston, R.H.W., 2006. Assessing the impacts of wind farms on birds. Ibis 148: 29-42.

Drewitt, A.L., Langston, R.H.W., 2008. Collision effects of wind-power generators and other obstacles on birds. Annals of New York Academy of Sciences 1134: 233-236.

Eastwood, E., 1967. Radar Ornithology. Methuen Press, London.

Edrén, S.M.C., Andersen, S.M., Teilmann, J., Carstensen, J., Harders, P.B., Dietz, R., Miller, L.A., 2010. The effect of a large Danish offshore wind farm on harbor and gray seal haul-out behavior. Marine Mammal Science 26: 614-634.

Edŕen, S.M.C., Teilmann, J., Dietz, R., Carstensen, J., 2004. Effects from the Construction of Nysted Offshore Wind Farm on Seals in Rødsand Seal Sanctuary Based on Remote Video Monitoring. Technical report to Energy E2 A/ S. National Environmental Research Institute, Roskilde.

Emu, Ltd, 2006. Kentish Flats Windfarm Development: Macrobenthic ecology study - 2005; Final Report 2006. Southampton, UK. 270 pp.

Engas, A., Lokkeborg, S., Ona, E., Soldal, A.V., 1996. Effects of seismic shooting on local abundance and catch rates of cod (Gadus morhua) and haddock (Melanogrammus aeglefinus). Canadian Journal of Fisheries and Aquatic Sciences 53: 2238-2249.

ESS Group, Inc., 2011. Cape Wind Energy Project: Construction and Operations Plan, Appendix H: Selected Regulatory Permits and Approvals and Correspondence. Cape Wind Associates, LLC. 163 pp.

Evans, W.R., Rosenburg, K.V., 2000. Acoustic monitoring of night-migrating birds: a progress report, in: Bonney Jr., R.E., Pashley, D.N., Cooper, R. (Eds), Strategies for Bird Conservation: Creating the Partners in Flight Planning Process. Proc. 3rd Partners in Flight Workshop; 1995 October 1-5; Cape May, NJ. Proc. RMRS-P-16. Ogden, UT: U.S. Dep. Agr. Forest Service, Rocky Mountain Research Station.

Everaert, J., Stienen, E.W.M., 2008. Impact of wind turbines on birds in Zeebrugge (Belgium). Biodiversity and Conservation in Europe 7: 103-117.

Exo, K.M., Hüppop, O., Garthe. S., 2003. Birds and offshore wind farms: a hot topic in marine ecology. Wader Study Group Bulletin 100: 50-53.

Fabi, G., Grati, F., Puletti, M., Scarcella, G., 2004. Effects on fish community induced by installation of two gas platforms in Adriatic Sea. Marine Ecology Progress Series 273: 187-197.

Farnsworth, A., 2005. Flight-call and their value for future ornithological studies and conservation research. Auk 122: 733-746

Farnsworth, A., Russell, R.W., 2005. Evaluation of a method for monitoring audible fall bird migration traffic over platforms. Pages 247-256. In: Interactions Between Migrating Birds and Offshore Oil and Gas Platforms in the Northern Gulf of Mexico. R.W. Russell, Ed. Mineral Management Service publication 2005-009.

Farnsworth, A., Russell, R.W., 2007. Monitoring flight calls of migrating birds from an oil platform in the Gulf of Mexico. Journal of Field Ornithology 78: 279-289.

Farnsworth, A., Gauthreaux, Jr., S.A., Van Blaricom, D., 2004. A comparison of nocturnal call counts of migrating birds and reflectivity measurements on Doppler radar (WSR-88D). Journal of Avian Biology 35: 365-369.

Fechhelm, R.G., Griffiths, W.B., Haley, B.E., Link, M.R., 2004. Nearshore Beaufort Sea Fish Monitoring in the Prudhoe Bay Region, 2003. Report for BP Exploration (Alaska) Inc. by LGL Alaska Research Associates, Inc., Anchorage, Alaska. 75 pp.

FERC (Federal Energy Regulatory Commission), 2008. Licensing Hydrokinetic Pilot Projects. FAQs. April 14, 2008. Federal Energy Regulatory Commission, Washington, DC.

FERC, 2006., Title 18: Conservation of Power and Water Resources Chapter 1: Federal Energy Regulatory Commission, Department of Energy, Part 5: Integrated license application process. http://www.access.gpo.gov/nara/cfr/waisidx_06/18cfr5_06.html; Last accessed 7/10/11.

FERC, 2010. Reedsport OPT Wave Park Settlement Agreement. FERC No. 12713, Douglas County, Oregon. 263 pp.

Ferguson, M.C., Barlow, J., Fiedler, P., Reilly, S.B., Gerrodette, T., 2006a. Spatial models of delphinid (family Delphinidae) encounter rate and group size in the eastern tropical Pacific Ocean. Ecological Modeling 193: 645-662.

Ferguson, M.C., Barlow, J., Reilly, S.B., Gerrodette, T., 2006b. Predicting Cuvier's (Ziphius cavirostris) and Mesoplodon beaked whale population density from habitat characteristics in the eastern tropical Pacific Ocean. Journal of Cetacean Research and Management 7: 287–299.

Fernandez-Juricic, E., 2001. Avian spatial segregation at edges and interiors of urban parks in Madrid, Spain. Biodiversity and Conservation 10: 1303–1316.

Fernie, K. J., Bird, D.M., Dawson, R.D., Lague, P.C., 2000. Effects of electromagnetic fields on the reproductive success of American Kestrels. Physiological and Biochemical Zoology 73: 60-65.

Firestone, J., Kempton, W., Krueger, A., Loper, C.E., 2005. Regulating offshore wind power and aquaculture: messages from land and sea. Cornell Journal of Law and Public Policy 14: 71-111.

Florida Fish and Wildlife Conservation Commission, 2006. Draft: Guidelines for the protection of marine animals during the use of explosives in the waters of the state of Florida. Florida Fish and Wildlife Conservation Commission, Tallahassee, FL.

Forman, R.T.T., Deblinger, R.D., 2000. The ecological road-effect zone of a Massachusetts (USA) suburban highway. Conservation Biology 14: 36–46.

Forney, K.A., 2000. Environmental models of cetacean abundance: reducing uncertainty in population trends. Conservation Biology 14: 1271-1286.

Fox, A.D., Desholm, M., Kahlert, J., Christensen, T.K., Petersen, I.K., 2006. Information needs to support environmental impact assessment of the effects of European marine offshore wind farms on birds. Ibis 148: 129-144.

Frid, C.L.J., 2011. Temporal variability in the benthos: Does the sea floor function differently over time? Journal of Experimental Marine Biology and Ecology 400: 99-107.

Frisk, G., Bradley, D., Caldwell, J., D'Spain, G., Gordon, J., Hastings, M., Ketten, D., Miller, J., Nelson, D.L., Popper, A.N., Wartzok, D., 2003, Ocean Noise and Marine Mammals, National Academy Press, Washington, D.C.

Fristedt, T., Moren, P., Soderberg, P., 2001. Acoustic and Electromagnetic Noise Induced by Wind Mills: Implications for Underwater Surveillance Systems Pilot Study. Report No. FOI-R-233-SE. Swedish Defence Research Agency, Stockholm, Sweden.

Garthe, S., Hüppop, O., 1999. Effect of ship speed on seabird counts in areas supporting commercial fisheries. Journal of Field Ornithology 70: 28-32.

Garthe, S., Hüppop, O., 2004. Scaling possible adverse effects of marine wind farms on seabirds: developing and applying a vulnerability index. Journal of Applied Ecology 41: 724-724.

Gauthreaux, S.A., Belser, C.G., 2006. Effects of artificial night lighting on migrating birds. In: Rich, C., Longcore, T. (Eds.), Ecologicial Consequences of Artificial Night Lighting. Island Press, Washington, DC, pp. 67–93.

Geomarine, Inc., 2010. Ocean/wind power ecological baseline studies; January 2008 – December 2009, Volume II: Avian Studies. New Jersey Department of Environmental Protection, Office of Science, New Jersey.

Gill, A.B., 2005. Offshore renewable energy: Ecological implications of generating electricity in the coastal zone. Journal of Applied Ecology 42: 605-615.

Gill, A.B., Gloyne-Phillips, I., Neal, K.J., Kimber, J.A., 2005. COWRIE 1.5 Electromagnetic Fields Review: The Potential Effects of Electromagnetic Fields Generated by Sub-Sea Power Cables Associated with Offshore Wind Farm Developments on Electrically and Magnetically Sensitive Marine Organisms—A Review. COWRIE Ltd. (Collaborative Offshore Wind Energy Research into the Environment) EM Field- 06-2004, 128 pp. Available online at: http:// www.thecrownestate.co.uk/newscontent/92_ electromagnetic_fields_phase_1.5

Gill, A.B., Gloyne-Phillips, I., Neal, K.J., Kimber, J.A., 2005. The potential effects of electromagnetic fields generated by sub-sea power cables associated with offshore wind farm developments on electrically and magnetically sensitive marine organisms - a review. COWRIE, Cranfield University, CMACS (Center for Marine And Coastal Studies, Ltd).

Gill, A.B., Huan, Y., Gloyne-Phillips, I., Metcalfe, J., Quayle, V., Spencer, J., Wearmouth, V., 2009. EMF-sensitive fish response to EM emissions from sub-sea electricity cables of the type used by the offshore renewable energy industry. COWRIE, Cranfield University, CEFAS, CIMS, CMACS (Center for Marine And Coastal Studies, Ltd).

Gill, J.P., Kemsley, C., Sales, D., 2002. The potential ornithological impact of the proposed Kentish Flats offshore wind farm. Report to GREP UK Marine Ltd., UK.

Gould, J.L., 2008. Animal navigation: the evolution of magnetic orientation. Current Biology 18: R482–R484.

Gould, P.J., Forsell, D.J., 1989. Techniques for shipboard surveys of marine birds. USDI, Fish and Wildlife Service Technical Report 25, Washington, D.C.

Grecian, W.J., Inger, R., Attrill, M.J., Bearhop, S., Godley, B.J., Witt, M.J., Votier, S.C., 2010. Potential impacts of wave-powered marine renewable energy installations on marine birds. Ibis 152: 683-697.

Griffin, L., Rees, E., Hughes, B., 2010. The migration of Whooper Swans in relation

to offshore wind farms. The Wildfowl and Wetlands Trust for COWRIE Ltd.

Grift, R.E., Couperus, A.S., Ybema, M.S., 2003. Base line studies North Sea wind farms: strategy of approach for pelagic fish. RIVO-Netherlands Institute for Fisheries Research. Report RKZ-1273. 20 pp.

Grift, R.E., Tien, N., 2003. Strategy of Approach Demersal Fish Fauna. RIKZ report 9M9237.01. 16 pp.

Hamilton, P.K., Knowlton, A.R., Marx, M.K., 2007. Right whales tell their own stories: the photo-identification catalog. In: Kraus, S.D., Rolland, R.M. (Eds.), The Urban Whale: North Atlantic Right Whales at the Crossroads. Harvard University Press, Cambridge, MA, pp. 75-104.

Hartung, R., 1995. Assessment of the potential for long-term toxicological effects of the Exxon Valdez oil spill on birds and mammals. In: Wells, P.G., Butler, J.N. (Eds.), Exxon Valdez oil spill: fate and effects in Alaskan waters. American Society for testing materials, Philadelphia, Pennsylvania, pp. 693-726

Hatch, J.J., Brault, S., 2007. Collision mortalities at Horseshoe Shoal of bird species of special concern. Report No. 5.3.2-1, Cape Wind Associates, Massachusetts.

Heinig, C.S., Tarbox, B.P., 2000. Pre- and post-dredge survey and relocation of the American lobster, Homarus americanus, population in Portland Harbor, Maine. Portland Harbor Dredge Committee and Casco Bay Estuary Project.

Henfridsson, U., Neimane, V., Strand, K., Kapper, R., Bernhoff, H., Danielsson, O., Leijon, M., Sundberg, J., Thorburn, K., Ericsson, E., Bergman, K., 2007. Wave energy potential in the Baltic Sea and the Danish part of the North Sea, with reflections on the Skagerrak. Renewable Energy 32: 2069–2084.

Hexter, R., 2009. High resolution video survey of seabirds and mammals in the Maray Firth, Hastings, West Isle of Wight and Bristol Channel Areas in periods 5,6 and 7. COWRIE Ltd., United Kingdom.

Hille Ris Lambers, R., ter Hofstede, R., 2009. Refugium Effects of the MEP-NSW Windpark on Fish: Progress Report 2007. Institute for Marine Resources and Ecosystem Studies (IMARES). 23 pp.

Hoffman, E., Astrup, J., Larsen, F., Munch-Petersen, S., 2000. Effects of Marine Windfarms on the Distribution of Fish, Shellfish and Marine Mammals in the Horns Rev Area. Report to ELSAMPROJEKT A/S. Report No. Baggrundsrapport 24. Danish Institute for Fisheries Research, Lyngby, Denmark.

Horn, J.W., Arnett, E.B., Kunz, T.H., 2008. Behavioral responses of bats to operating wind turbines. Journal of Wildlife Management 72: 123-132.

Horowitz, C., Jasny, M., 2007. Precautionary management of noise: lessons from the U.S. Marine Mammal Protection Act. Journal of International Wildlife Law and Policy 10: 225–232.

Hötker, H., Thomsen, K.M., Jeromin, H., 2006. Impacts on biodiversity of exploitation of renewable energy sources: the example of birds and bats – facts, gaps in knowledge, demands for further research, and ornithological guidelines for the development of renewable energy exploitation. Michael-Otto-Institut im NABU, Bergenhusen. http://bergenhusen.nabu.de/bericht/englische%20 windkraftstudie.pdf

Hubbard, H.H., Shepherd, K.P., 1990. Wind turbine acoustics. NASA Technical Paper 3057 DOE/NASA 20320-77. Hampton, Virginia.

Hüppop, O., Dierschke, J., Exo, K.M., Frederich, E., Hill, R., 2006. Bird migration studies and potential collision risk with offshore wind turbines. Ibis 148: 90-109.

Inger, R., Attrill, M.J., Bearhop, S., Broderick, A.C., Grecian, W.J., Hodgson, D.J., Mills, C., Sheehan, E., Votier, S.C., Witt, M.J., Godley, B.J., 2009. Marine renewable energy: potential benefits to biodiversity? An urgent call for research. Journal of Applied Ecology 46: 1145-1153.

Inger, R., M.J. Attrill, S. Bearhop, A. C. Broderick, W. J. Grecian, D. J. Hodgson, C. Mills, E. Sheehan, S. C. Votier, M. J. Witt, and B. J. Godley. 2009. Marine renewable energy: potential benefits to biodiversity? An urgent call for research.Journal of Applied Ecology 4:1145-1153.

Jarvis, C.M., 2005. An evaluation of the wildlife impacts of offshore wind development relative to fossil fuel power production. M.S. Thesis, University of Delaware, Graduate College of Marine Studies.

Jarvis, S., Allen, J., Proctor, N., Crossfield, A., Dawes, O., Leighton, A., McNeill, L., Musk, W., 2004. North Sea Wind Farms: NSW Lot 1 Benthic Fauna. Final Report. University of Hull. 91 pp.

JNCC, NE, & CCW (Joint Nature Conservation Commission, Natural England, and Countryside Council for Wales), 2010. The protection of marine European protected species from injury and disturbance: Guidance for the marine area in England and Wales and the UK offshore marine area. Joint Nature Conservation Commission, Natural England, and Countryside Council for Wales.

Johnson, J.B., Gates, J.E., Zegre, N.P., 2010. Monitoring seasonal bat activity on a coastal barrier island in Maryland, USA. Environmental Monitoring and Assessment 173: 685-689.

Jonkman, J., Butterfield, S., Musial, W., Scott, G., 2009. Definition of a 5-MW reference wind turbine for offshore system development. National Renewable Energy Laboratory Tech. Report NREL/TP_500_38060.

Golden, Colorado.
Kahlert, J., Petersen, I.K., Fox, A.D., Desholm, M., Clausager, I., 2004. Investigations of birds during construction and operation of Nysted offshore wind farm at Rodsand. Annual status report 2003. NERI report. Kalo, Denmark: National Environmental Research Institute. 82pp.
Kahlert, J., Petersen, I.K., Desholm, M., 2007. Effects on birds of the Rødsand 2 offshore wind farm: Environmental Impact Assessment. NERI, University of Aarhus, Denmark.
Kahlert, J., Desholm, M., Clausager, I., Petersen, I.K., 2000. Environmental impact assessment of an offshore wind park at Rodsand: Technical report on birds. NERI, Denmark.
Kaiser, M.J., Galanidi, M., Showler, D.A., Elliot, A.J., Caldow, R.W.G., Rees, E.I.S., Stillman, R.A., Sutherland, W.J., 2006. Distribution and behavior of common scoter Melanitta nigra relative to prey resources and environmental parameters. Ibis 148: 110-128.
Kamperman, G.W., James, R.R., 2008. The "how to" guide to siting wind turbines to prevent health risks from sound. Available online: http://www.crge.org/doc/kamperman-james-10-28-08.pdf
Katranitsas, A., Castritsi-Catharios, J., Persoone, G., 2001. The effects of a copper-baed antifouling paint on mortality and enzymatic activity of a non-target organism. Marine Pollution Bulletin 46: 1491-1494.
Keevin, T.M., Hempen, G.L., 1997. The environmental effects of underwater explosions with methods to mitigate impacts. U.S. Army Corps of Engineers, St. Louis, MO.
Kelly, T.A., West, T.E., Davenport, J.K., 2009. Challenges and solutions of remote sensing at offshore wind energy developments. Marine Pollution Bulletin 58: 1599-1604.
Kenney, R.D., Shoop, C.R., In press. Aerial surveys for marine turtles. In: McDiarmid, R.W, Foster, M.S., Gibbons, J.W., Guyer, C., Chernoff, N. (Eds.). Sampling Reptile Diversity: Standard Methods for Inventory and Monitoring. University of California Press, Berkeley, CA, pp. 264-271
Kerlinger, P., Gehring, J.L., Erickson, W.P., Curry, R., Jain, A., Guarnaccia, J., 2010. Night migrant fatalities and obstruction lighting at wind turbines in North America. The Wilson Journal of Ornithology 122(4): 744–754.
King, S., Maclean, I., Norman, T., Prior, A., 2009. Developing guidance on ornithological cumulative impact assessment for offshore wind developers. COWRIE UK.
Kinney, J., Flood, R.D., 2005. Seabed morphology off Southern Long Island: Studies of artificial reefs and implications for wind farms. Marine Sciences Research Center, Stony Brook University. 14 pp.

Kirshvink, J.L., 1997. Magnetoreception: Homing in on vertebrates. Nature 690: 339–340.

Klima, E.F., Gitschlag, G.R., Renaud, M.L., 1988. Impacts of the explosive removal off offshore petroleum platforms on sea turtles and dolphins. Marine Fisheries Review 50(3): 33-42.

Komdeur, J., Bertelsen, J., Cracknell, G., 1992. Manual for aeroplane and ship survyes of waterfowl and seabirds. International Waterfowl and Wetlands Bureau (IWRB) Special Publication no. 19. Ronde, Denmark: National Environmental Research Institute.

Kostylev, V.E., Hannah, C.G., 2007. Process-driven characterization and mapping of seabed habitats. In: Todd, B.J., Greene, H.G. (Eds.), Mapping the seafloor for habitat characterization: Geological Association of Canada. Geological Association of Canada, pp. 171-184.

Krägefsky, S., 2010. Project DEFI-av (StUKplus): Study of the effects of offshore wind farms on fish and megafauna. In: Environmental research results at alpha ventus - first results. Bundesamt für Seeschifffahrt und Hydrographie (BSH).

Kraus, S. D., 1990. Rates and potential causes of mortality in North Atlantic right whales (Eubalaenaglacialis). Marine Mammal Science 6: 278–291.

Krijgsveld, K.L., Akershoek, K., Schenk, F., Dijk, F., Dirksen, S., 2010. Collision risk of birds with modern large wind turbines. Ardea 97: 357-366.

Kunz, T.H., Arnett, E.B., Cooper, B.M., Erickson, W.P., Larkin, R.P., Mabee, T., Morrison, M.L., Strickland, M.D., Szewczak, J.M., 2007. Assessing impacts of wind-energy development on nocturnally active birds and bats: a guidance document. Journal of Wildlife Management 71: 2449-2486.

Kwan, K., Ho, C., Mei, G., Li, Y., Ren, Z., Xu, R., Zhang, Y., Lao, D., Stevenson, M., Stanford, V., Rochet, C., 2006. An Automated Acoustic System to Monitor and Classify Birds, EURASIP 2006: 1-19.

Langhamer, O., Wilhelmsson, D., 2009. Colonization of fish and crabs of wave energy foundations and the effects of manufactured holes: A field experiment. Marine Environmental Research 68: 151–157.

Langhammer, O., Wilhelmsson, D., Engstrom, J., 2009. Artificial reef effect and fouling impacts on offshore wave power foundations and buoys - a pilot study. Estuarine, Coastal, and Shelf Science 82: 426-432.

Langston, R.H.W., Pullan, J.D., 2003. Wind farms and birds: an analysis of the effects of wind farms on birds, and guidance on environmental assessment criteria and site selection issues. Report written by Birdlife International on behalf of the Bern Convention. Council Europe Report T-PVS/ Inf.

Langton, R., Davies, I.M., Scott, B.E., 2011. Seabird conservation and tidal stream

and wave power generation: Information needs for predicting and managing potential impacts. Marine Policy 35: 623-630.
Larkin, R.P., Evans, W.R., Diehl, R.H., 2002. Nocturnal flight call of Diskcissels and doppler radar echoes over south Texas in spring. Journal of Field Ornithology 73: 2-8.
Larsen, J.K., Guillemette, M., 2007. Effects of wind turbines on flight behaviour of wintering common eiders: Implications for habitat use and collision risk. Journal of Applied Ecology 44: 516–522.
Leddy, K.L., Higgins, K.F., Naugle, D.E., 1999. Effects of wind turbines on upland nesting birds in conservation reserve program grasslands. Wilson Bulletin 111: 100-104.
Lefebvre, A., Thompson, C.E.L., Collins, K.J., Amos, C.L., 2009. Use of a high-resolution profiling sonar and a towed video camera to map a Zostera marina bed, Solent, UK. Estuarine, Coastal and Shelf Science 82: 323-334.
Leighton, F.A., 1990. The toxicity of petroleum oils to birds: an overview. In: Proceedings of the Effects of Oil on Wildlife, Oil Symposium 1990, Oct 1990, Herndon, VA, pp. 10-22.
Leijon, M., Bernhoff, H., Berg, M., Ågren, O., 2003. Economical considerations of renewable electric energy production – especially development of wave energy. Renewable Energy 28: 1201–1209.
Leonhard, S., Birklund, O., Birklund, J., Environment, D.W. and, 2006. Danish offshore wind: Key Environmental Issues; Chapter 6: Infauna, epifauna and vegetation - Change in diversity and higher biomass. DONG Energy, Vattenfall, The Danish Energy Authority, and the Danish Forest and Nature Agency. pp 44-63.
Logerwell, R., 2008. Beaufort Sea Marine Fish Monitoring 2008: Pilot Survey and Test of Hypotheses. Alaska Fisheries Science Center, NOAA, Seattle, WA.
Longcore, T., Rich, C., Gauthreaux, S.A., 2008. Height, guy wires, and steady-burning lights increase hazard of communication towers to nocturnal migrants: a review and meta-analysis. Auk 125: 485-492.
Louzao, M., Becares, J., Rodriquez, B., Hyrenback, K.D., Ruiz, A., Arcos, J.M., 2009. Combining vessel-based surveys and tracking data to identify key marine areas for seabirds. Marine Ecology Progress Series 391: 183-197.
Love, M.S., York, A., 2005. A Comparison of the fish assemblages associated with an oil/gas pipeline and adjacent seafloor in the Santa Barbara Channel, Southern California Bight. Bulletin of Marine Science 77 (1): 101-117.
Love, M.S., Caselle, J., Snook, L., 1999. Fish Assemblages on mussel mounds surrounding seven oil platforms in the Santa Barbara Channel and Santa Maria Basin. Bulletin of Marine Science 65 (2): 497-513
Love, M.S., Hyland, J., Ebeling, A., Herrlinger, T., Brooks, A., Imamura, E., 1994. A

pilot study of the distribution and abundances of rockfishes in relation to natural environmental factors and an offshore oil and gas production platform off the coast of Southern California. Bulletin of Marine Science 53 (2-3): 1062-1085.

Luschi, P., Benhamou, S., Girard, C., Ciccione, S., Roos, D., Sudre, J., Benvenuti, S., 2007. Marine turtles use geomagnetic cues during open-sea homing. Current Biology 17: 126–133.

Maclean, I.M.D., Skov, H., Rehfisch, M.M., Piper, W., 2006. Use of aerial surveys to detect bird displacement by offshore windfarms. COWRIE DIPS-03-2006, BTO Research Report No. 446, UK.

Maclean, I.M.D., Skov. H., Rehfisch, M.M., 2007. Further use of aerial surveys to detect bird displacement by offshore windfarms. COWRIE Ltd, Cumbria, United Kingdom.

Maclean, I.M.D., Wright, L.J., Showler, D.A., Rehfisch, M.M., 2009. A review of assessment methodologies for offshore wind farms. British Trust for Ornithology, COWRIE Ltd., United Kingdom.

Madsen, P.T., Wahlberg, M., Tougaard, J., Lucke, K., Tyack, P., 2006. Wind turbine underwater noise and marine mammals: implications of current knowledge and data needs. Marine Ecology Progress Series 309: 279-295.

Mann, R., Harding, J.M., Southworth, M.J., Wesson, J.A., 2005. Northern quahog (hard clam) Mercenaria mercenaria abundance and habitat use in Chesapeake Bay. Journal of Shellfish Research 24(2): 509-516.

Martec Limited, 2004. Effects of pipelines/gathering lines on snow crab and lobster. Martec Limited, CEF Consultants Ltd, DRDC Atlantic, St. Francis Xavier University, Calgary.

Masden, E.A., Haydon, D.T., Fox, A.D., Furness, R.W., Bullman, R., Desholm, M., 2010. Barriers to movement: Modelling energetic costs of avoiding marine wind farms amongst breeding seabirds. Marine Pollution Bulletin 60: 1085-1091.

Masden, E.A., Haydon, D.T., Fox, A.D., Furness, R.W., Bullman, R., Desholm, M., 2009. Barriers to movement: Impacts of wind farms on migrating birds. ICES Journal of Marine Science 66: 746–753.

Meibner, K., Sordyl, H., 2006. Ecological research on offshore wind farms: International exchange of experiences; Chapter 1: Literature review of offshore wind farms with regard to benthic communities and habitats. German Federal Agency for Nature Conservation. pp 1-46.

Mellor, M., Maher, M., 2008. Full Scale Trial of High Definition Video Survey for Offshore Windfarm Sites. COWRIE Ltd., Whitehaven, England.

Mellor, M., Craig, T., Baillie, D., Woolaghan, P., 2007. Trial High Definition Video Survey of Seabirds. COWRIE Ltd., Whitehaven, England.

Miller, J.H., Potty, G.R., Vigness Raposa, K., Casagrande, D., Miller, L., Crocker, S., Tyce, R., Preston, J., Roderick, B., Nystuen, J., Scheifele, P., 2009. Environmental assessment of offshore wind power generation near Rhode Island: acoustic and electromagnetic effects on marine animals. Presentation, Acoustical Society of America meeting, San Antonio, Texas.

MMS (Minerals Management Service), 2001. Development and Design of Biological and Physical Monitoring Protocols to Evaluate the Long-term Impacts of Offshore Dredging Operations on the Marine Environment. MMS International Activities and Marine Minerals Division. MMS 2001-089.

MMS, 2008. Framework for the avian and bat monitoring plan for the Cape Wind Proposed Offshore Wind Facility. Appendix N. MMS, Herdon, VA.

MMS, 2008b. Long-Term Monitoring at the East and West Flower Garden Banks, 2004-2005 - Interim Report.

MMS, 2009. Cape Wind Energy Project; Final Environmental Impact Statement. MMS EIS-EA, OCS Publication No. 2008-40, U.S. Dept. of the Interior, Minerals Management Service, Herndon, VA.

Mizrahi, D., Fogg, R., Margarian, T., Elia, V., La Puma, D., 2010. Radar monitoring of bird and bat movement patterns on Block Island and its coastal waters. University of Rhode Island, Department of Natural Resource Science, 53 pp.

Moein-Bartol, S., Musick, J.A., 2003. Sensory biology of sea turtles. In: Lutz, P.L., Musick, J.A., Wyneken, J. (Eds.), The Biology of Sea Turtles, Volume II. Boca Raton, Florida: CRC Press, Inc., pp. 79-102

Montgomery, J., Carton, G., Voigt, R., Baker, C., Diebel, C., 2000. Sensory processing of water currents by fishes. Philosophical Transactions of the Royal Society of London B 355: 1325–1327.

Murawski, S.A., Wigley, S.E., Fogarty, M.J., Rago, P.J., Mountain, D.G., 2005. Effort distribution and catch patterns adjacent to temperate MPAs. ICES Journal of Marine Science 62: 1150–1167.

Muxika, I., Borja, A., Bald, J., 2007. Using historical data, expert judgement and multivariate analysis in assessing reference conditions and benthic ecological status, according to the European Water Framework Directive. Marine Pollution Bulletin 55: 16-29.

Muxika, I., Ibaibarriaga, L., Saiz, J., Borja, A., 2007. Minimal sampling requirements for a precise assessment of soft-bottom macrobenthic communities, using AMBI. Journal of Experimental Marine Biology and Ecology 349: 323-333.

NRC (National Research Council), 2007. Environmental impacts of wind-energy projects. The National Academies Press, Washington, DC.

Nedwell, J., Howell, D., 2004. A review of offshore windfarm related underwater noise sources. COWRIE Technical Report 544 R 0308, The Crown Estate, London, U.K.
Nedwell, J., Langworthy, J., Howell, D., 2004. Assessment of sub-sea acoustic noise and vibration from offshore wind turbines and its impact on marine wildlife; initial measurements of underwater noise during construction of offshore windfarms, and comparison with background noise. COWRIE report N. 544 R 0424. The Crown Estate, London, U.K.
Nedwell, J., Langworthy, J., Howell, D., 2003. Initial measurements of underwater noise during construction of offshore windfarms, and comparison with background noise, Assessment of sub-sea acoustic noise and vibration from offshore wind turbines and its impact on marine wildlife. Scubacoustech, Report no. 544 R 0424, 72 pp.
Nicholls, B., Racey, P.A., 2007. Bats avoid radar installations: could electromagnetic fields deter bats from colliding with wind turbines? Plos One 2: e297: 1-6.
NMFS (U.S. National Marine Fisheries Service), 2003. Taking of marine mammals incidental to specified activities; Construction of the east span of the San Francisco-Oakland Bay Bridge; Notice of issuance of an incidental harassment authorization. Federal Register 68(220): 54595-64609.
NMFS, 2009a. Department of Commerce, National Oceanic and Atmospheric Administration, National Marine Fisheries Service, Letter of Authorization. National Marine Fisheries Service, Office of Protected Resources, Silver Spring, MD.
NMFS, 2009b. Taking and importing marine mammals; U.S. Navy training in the Cherry Point Range Complex; Final Rule. Federal Register 74(113): 28370-28391.
NMFS, 2009c. Taking and importing marine mammals; U.S. Navy training in the Hawaii Range Complex; Final Rule. Federal Register 74(7): 1456-1491.
NMFS, 2009d. Taking and importing marine mammals; U.S. Navy training in the Jacksonville Range Complex; Final Rule. Federal Register 74(113): 28349-28370.
NMFS, 2009e. Taking and importing marine mammals; U.S. Navy training in the Southern California Range Complex; Final Rule. Federal Register 74(12): 3883-3918.
NMFS, 2009f. Taking and importing marine mammals; U.S. Navy training in the Virginia Capes Range Complex; Final Rule. Federal Register 74(113): 28328-28349.
NMFS, 2009g. Taking and importing marine mammals; U.S. Navy's Atlantic Fleet Active Sonar Training (AFAST); Final Rule. Federal Register 74(16):

4844-4885.

NMFS, 2010a. Takes of marine mammals incidental to specified activities; Manette Bridge replacement in Bremerton, Washington; Notice; issuance of an incidental harassment authorization. Federal Register 75(128): 38783-38791.

NMFS, 2010b. Takes of marine mammals incidental to specified activities; Taking marine mammals incidental to open water marine seismic survey in the Chukchi Sea, Alaska; Notice; issuance of an incidental harassment authorization. Federal Register 75(156): 49760-49811.

NMFS, 2010c. Takes of marine mammals incidental to specified activities; Taking marine mammals incidental to operation and maintenance of a liquefied natural gas facility off Massachusetts; Notice; issuance of an incidental harassment authorization. Federal Register 75(136): 41440-41449.

NMFS, 2010d. Takes of marine mammals incidental to specified activities; Taking marine mammals incidental to operations of a liquefied natural gas port facility in Massachusetts Bay; Notice of issuance of an incidental harassment authorization. Federal Register 75(169): 53672-53678.

NMFS, 2010e. Taking and importing marine mammals; Military training activities and research, development, testing and evaluation conducted within the Marianas Islands Range Complex (MIRC); Final Rule. Federal Register 75(148): 45527-458556.

NMFS, 2010f. Taking and importing marine mammals; Navy training activities conducted within the Northwest Training Range Comples (NWTRC) and military training activities and research, development, testing and evaluation conducted witht the Mariana Island Range Complex (MIRC); Notice; issuance three letters of authorization. Federal Register 75(243): 79342-79344.

NMFS, 2010g. Taking and importing marine mammals; Navy training activities conducted within the Northwest Training Range Complex; Final Rule. Federal Register 75(217): 69296-69326.

NMFS, 2010h. Taking and importing marine mammals; Taking marine mammals incidental to Navy operations of Surveillance Towed Array Sensor System Low Frequency Active Sonar; Notice; issuance of three Letters of Authorization. Federal Register 75(161): 51443-51444.

NMFS, 2010i. Taking and importing marine mammals; Taking marine mammals incidental to Navy training exercises in there East Coast Range Complexes; Notice; issuance of three letters of authorization. Federal Register 75(116): 34428-34429.

NMFS, 2010j. Taking and importing marine mammals; U.S. Navy training in the Hawaii Range Complex; Notice of issuance of a letter of authorization.

Federal Register 75(16): 4049-4051.
NMFS, 2010k. Taking and importing marine mammals; U.S. Navy training in the Southern California Range Complex; Notice of issuance of a letter of authorization. Federal Register 75(16): 4047-4049.
NMFS, 2010l. Taking and importing marine mammals; U.S. Navy's Atlantic Fleet Active Sonar Training; Notice of issuance of a letter of authorization. Federal Register 75(20): 5055-5056.
Noer, H., Christensen, T.K., Clausager, I.B., Petersen, I.K., 2000. Effects on birds of an offshore wind park at Horns Rev: Environmental impact assessment. NERI, Denmark
Norman, T., Buisson, R., Askew, N., 2007. COWRIE workshop on the cumulative impact of offshore windfarms on birds. COWRIE CIBIRD-01-2007, Peterborough, UK.
Nowacek, D.P., Thorne, L.H., Johnson, D.W., Tyack, P.L., 2007. Responses of cetaceans to anthropogenic noise. Mammal Review 37: 81–115.
NRC (National Research Council), 1990. Decline of the Sea Turtles: Causes and Prevention. National Academy Press, Washington, DC.
NRC, 2000. Marine Mammals and Low-Frequency Sound: Progress Since 1994. National Academy Press, Washington, DC.
NRC, 2005. Marine Mammal Populations and Ocean Noise: Determining When Noise Causes Biologically Significant Effects. National Academies Press, Washington, DC.
NWCC (National Wind Coordinating Committee), 2004. Wind turbine interactions with birds and bats: a summary of research results and remaining questions. National Wind Coordinating Committee, Nov. 2004. Available at www.nationalwind.org.
NWP Offshore Limited, 2008. North Hoyle Offshore Wind Farm - Final Annual FEPA Monitoring Report and Five Year Monitoring Program Summary. Npower Renewables Limited, Berkrshire, UK.
O'Hara, J., Wilcox, J.R., 1990. Avoidance responses of loggerhead turtles, Caretta caretta, to low frequency sound. Copeia 1990: 564-567.
Ogden, L.J.E., 1996. Collision course: the hazards of lighted structures and windows to migration birds. Report to WWF Canada and the Fatal Light Awareness Program. http://www.flap.org/new/ccourse.pdf
Ohman, M.C., Sigray, P., Westerberg, H., 2007. Offshore Windmills and the Effects of Electromagnetic Fields on Fish. Ambio 36(8): 630-633.
Orpin, A., Kostylev, V., 2006. Towards a statistically valid method of textural sea floor characterization of benthic habitats. Marine Geology 225: 209-222.
OSPAR Commission, 2002. Joint Assessment and Monitoring Programme (JAMP) guidelines on quality assurance for biological monitoring in the OSPAR

area. Ref no. 2002-15, 38 pp.
OSPAR Commission, 2004. Problems and Benefits Associated With the Development of Offshore Wind-Farms. Publication 212/2004. OSPAR Convention for the Protection of the Marine Environment of the North-East Atlantic.
OSPAR Commission, 2006. Review of the Current State of Knowledge on the Environmental Impacts of the Location, Operation and Removal/Disposal of Offshore Wind-Farms; Status Report April 2006. Publication 278/2006. OSPAR Convention for the Protection of the Marine Environment of the North-East Atlantic.
OSPAR Commission, 2008. Assessment of the Environmental Impact of Offshore Wind-Farms. Publication 385/2008. OSPAR Convention for the Protection of the Marine Environment of the North-East Atlantic.
OSPAR Commission, 2009. Overview of the Impacts of Anthropogenic Underwater Sound in the Marine Environment. Publication 441/2009. OSPAR Convention for the Protection of the Marine Environment of the North-East Atlantic.
Palsson, W.A., Tsou, T.S., Bargmann, G.G., Buckley, R.M., West, J.E., Mills, M.L., Cheng, Y.W., Pacunski, R.E., 2009. The Biology and Assessment of Rockfishes in Puget Sound. Washington Department of Fish and Wildlife: Fish Program.
Pandey. A., Hermence, J., Harness, R., 2007. Development of a cost-effective system to monitor wind turbines for bird and bat collisions. Phase 1: sensor system feasibility study. California Energy Commission, CEC-t00-2007-004.
Paton, P., Winiarski, K.J., Trocki, C.L., McWilliams, S.R., 2010. Spatial distribution, abundance and flight ecology of birds in nearshore and offshore waters of Rhode Island. RI Ocean SAMP, Technical report #11, pp. 971-1274.
Pauly, D., Christensen, V., Dalsgaard, J., Froese, R., Torresh, Jr., F., 1998. Fishing down marine food webs. Science 279: 860–863.
PCAD Working Group, 2010. Introduction and progress of a working group on the population consequences of acoustic disturbance. IWC Scientific Committee meeting document SC/62/WW5. International Whaling Commission, Cambridge, UK.
Peabody, M.B., Wilson, C.A., 2006. Fidelity of Red Snapper (Lutjanus campechanus) to Petroleum Platforms and Artificial Reefs in the Northern Gulf of Mexico. U.S. Minerals Management Service, Washington, DC.
Pearson, T.H., Rosenberg, R., 1978. Macrobenthic succession in relation to organic enrichment and pollution of the marine environment.

Oceanography and Marine Biology: An Annual Review 16: 229-311.
Pedersen E., Persson, W.K., 2004. Perception and annoyance due to wind turbine noise – a dose-response relationship. Journal of the Acoustical Society of America 116: 3460-3470
Pelc, R., Fujita, R.M., 2002. Renewable energy from the ocean. Marine Policy 26: 471-479.
Percival, S.M., 2001. Assessment of the effects of offshore wind in birds. DTA/Pub URN 01/1434. UK.
Percival, S.M., 2003. Birds and wind farms in Ireland: a review of potential issues and impact assessment. Unpubl. report. UK. 25 pp.
Perkins, S., Sadoti, G., Allison, T., Jones, A., 2004. Relative waterfowl abundance within Nantucket Sound, Massachusetts during the 2003-2004 winter season. Unpublished report, Massachusetts Audubon Society. Lincoln, Massachusetts.
Perrow, M.R., Skeate, E.R., Lines, P., Brown, D., Tomlinson, M.L., 2006. Radio telemetry as a tool for impact assessment of wind farms: the case of little terns Sterna albifrons at Scroby Sands, Norfolk, UK. Ibis 148: 57-75.
Petersen, I.K., 2005. Bird numbers and distributions in the Horns Rev offshore wind farm area. National Environmental Research Institute Report, Denmark.
Petersen, I.K., Christensen, C.K., Kahlert, J., Desholm, M., Fox, A.D., 2006. Final results of bird studies at the offshore wind farms at Nysted and Horns Rev, Denmark. NERI Report, commissioned by DONG Energy and Vattenfall A/S, DK 166.
Pettersson, J., 2005. The Impact of offshore wind farms on bird life in southern Kalmar Sound, Sweden. Lunds University, Sweden.
Pickrill, R.A., Kostylev, V E, 2007. Habitat mapping and national seafloor mapping strategies in Canada. In: Todd, B.J., Greene, H.G. (Eds.), Mapping the seafloor for habitat characterization: Geological Association of Canada. Geological Association of Canada, pp. 483-495.
Pilling, G.M., van der Kooij, J., Daskalov, G.M., Cotter, A.J.R., Metcalfe, J.D., 2007. Overview of current best world practice in fish stock assessment and management, with specific reference to Caspian Sea fisheries. Sci. Ser. Tech. Rep., Cefas Lowestoft, 158 pp.
Polagye, B., Copping, A., Kirkendall, K., Boehlert, G., Walker, S., Wainstein, M., van Cleve, B., 2010. Environmental Effects of Tidal Energy Development: A Scientific Workshop. Unpublished Workshop briefing paper. University of Washington, Seattle. Available online: http://depts.washington.edu/nnmrec/workshop/docs/Tidal_energy_briefing_paper.pdf.

Polagye, B., van Cleve, B., Copping, A., Kirkendall, K. (Eds.), 2011. Environmental effects of tidal energy development. U.S. Dept. Commerce, NOAA Tech. Memo. NMFS F/SPO-116. 186 pp.

Polish Wind Energy Association, 2008. Guidelines for assessment of wind farms' impact on birds. Szczecin. 26 http://www.fws.gov/windenergy/docs/Examples_State_International_Guidance_Duration_Intensity_Pre.pdf

Pollock, C.M., Mavor, R., Weir, C.R., Reid, A., White, R.W., Tasker, M.L., Webb, A., Reid, J.B., 2000. The distribution of seabirds and marine mammals in the Atlantic frontier, north and west of Scotland. Joint Nature Conservation Committee.

Poot, H., Ens, B.J., de Vries, H., Donners, M.A.H., Wernand, M.R., Marquenie, J.M., 2008. Green light for nocturnally migrating birds. Ecology and Society 13 (2): 47.

Popper, A.N., Hastings, M.C., 2009. The effects of anthropogenic sources of sound on fishes. Journal of Fish Biology 75: 455-489.

Portman, M., 2009. Involving the public in the impact assess of offshore renewable energy facilities. Marine Policy 33: 332-338.

Powers, K., 1983. Pelagic distributions of marine birds off the northeastern United States. NOAA Technical Memorandum.

Prior, A., McMath, M.J., 2008. Marine mammals and noise from offshore renewables projects -- UK developments. In: Evans, P.G.H. (Ed.), Proceedings of the ASCOBANS/ECS Workshop; Offshore Wind Farms and Marine Mammals: Impacts and Methodologies for Assessing Impacts. ECS Special Publication Series No. 49, European Cetacean Society, pp. 12-16.

Proctor, N., Jarvis, S., Allen, J., 2003. North Sea Windfarms; Lot 1 Benthic Fauna. Phase 1. Strategy of Approach. University of Hull. 20 pp.

Quintino, V., Elliott, M., Rodrigues, A., 2006. The derivation, performance and role of univariate and multivariate indicators of benthic change: Case studies at differing spatial scales. Journal of Experimental Marine Biology and Ecology 330: 368-382.

Rademacher, K.R., Render, J.H., 2003. Fish assemblages around oil and gas platforms in the Northeastern Gulf of Mexico: Developing a survey design. In: Fisheries, Reefs, and Offshore Development. Stanley, D.R., Scarborough-Bull, A. (Eds.), American Fisheries Society Symposium 36. Bethesda, Maryland: American Fisheries Society.

Ranke, J., 2002. Persistence of antifouling agents in the marine biosphere, Environmental Science and Technology 36: 1539–1545.

Redfern, J.V., Ferguson, M.C., Becker, E.A., Hyrenbach, K.D., Good, C., Barlow, J., Kaschner, K., Baumgartner, M.F., Forney, K.A., Ballance, L.T., Fauchald, P., Halpin, P., Hamazaki, T., Pershing, A.J., Qian, S.S., Read, A., Reilly, S.B.,

Torres, L., Werner, F., 2006. Techniques for cetacean-habitat modeling: a review. Marine Ecology Progress Series 310: 271-295
Rees, H.L., Hyland, J.L., Hylland, K., Mercer Clarke, C.S.L., Roff, J.C., Ware, S., 2008. Environmental indicators: utility in meeting regulatory needs. An overview. ICES Journal of Marine Science 65: 1381-1386.
Reiche, D., Bechberger, M., 2004. Policy differences in the promotion of renewable energies in the EU member states. Energy Policy 32: 843–9.
Reubens, J., Degraer, S., Vincx, M., 2010 The importance of marine wind farms, as artificial hard substrata, for the ecology of the ichthyofauna. In: Degraer, S., Brabant, R., Rumes, B. (Eds.), Offshore wind farms in the Belgian part of the North Sea: Early environmental impact assessment and spatio-temporal variability. Royal Belgian Institute of Natural Sciences, pp. 69-82.
Rhoads, D.C., McCall, P.L., Yingst, J.Y., 1978. Disturbance and production on the estuarine seafloor. American Scientist 66: 577-586.
Rice, J., Arvanitidis, C., Borja, A., Frid, C., Hiddink, J., Krause, J., Lorance, P., Ragnarsson, Á., Sköld, M., Trabucco, B., 2010. Marine Strategy Framework: Task Group 6 Report: Seafloor Integrity. European Commission Joint Research Centre. 81 pp.
Ridgway, S.H., Wever, E.G., McCormick, J.G., Palin, J., Anderson, J.H., 1969. Hearing in the giant sea turtle, Chelonia mydas. Proceedings of the National Academy of Sciences 64: 884-890.
Ross, B.P., Lien, J., Furness, R.W., 2001. Use of underwater playback to reduce the impact of eiders on mussel farms. ICES Journal of Marine Science 58: 517–524.
RPS Planning and Development, 2008. Gunfleet Sands Offshore Wind Farm Development. DONG Energy, Copenhagen, Denmark.
Rye, J.H., Gilles, A., Verfuß, U., 2008. Linking wind farms and harbor porpoises: review of methods. In: Evans, P.G.H. (Ed.), Proceedings of the ASCOBANS/ECS Workshop; Offshore Wind Farms and Marine Mammals: Impacts and Methodologies for Assessing Impacts. ECS Special Publication Series No. 49, European Cetacean Society, pp. 21-26.
Samhouri, J.F., Levin, P.S., Andrew James, C., Kershner, J., Williams, G., 2011. Using existing scientific capacity to set targets for ecosystem-based management: A Puget Sound case study. Marine Policy 35: 508-518.
Scheidat, M., Tougaard, J., Brasseur, S., Carstensen, J., van Polanen Petel, T., Teilmann, J., Reijnders, P., 2011. Harbor porpoises (Phocoena phocoena) and wind farms: a case study in the Dutch North Sea. Environmental Research Letters 6: 10 pp.
Schroeder, B.A., Thompson, N.B., 1987. Distribution of the loggerhead turtle,

Caretta caretta, and the leatherback turtle, Dermochelys coriacea, in the Cape Canaveral, Florida area: results of aerial surveys. In: Witzell, W.N. (Ed.), Ecology of East Florida Sea Turtles. Proceedings of the Cape Canaveral, Florida Sea Turtle Workshop, Miami, Florida, February 26-27, 1985. NOAA Technical Report NMFS 53. National Marine Fisheries Service, Miami, Florida, pp. 45-53.

SEAS, 2002. Possible effects of the offshore wind farm at Vindeby on the outcome of fishing. Bio/Consult A/S.

SeaScape Energy Ltd., 2002. Burbo Offshore Wind Farm, Vol 2: Environmental Statement. DONG Energy, Copenhagen, Denmark.

Sheehan, E.V., Stevens, T.F., Attrill, M.J., 2010. A quantitative, non-destructive methodology for habitat characterisation and benthic monitoring at offshore renewable energy developments. PloS one 5: e14461.

Shoop, C.R., Kenney, R.D., 1992. Seasonal distributions and abundances of loggerhead and leatherback sea turtles in waters of the northeastern United States. Herpetological Monographs 6: 43-67.

Slotte, A., Hansen, K., Dalen, J., Ona, E., 2004. Acoustic mapping of pelagic fish distribution and abundance in relation to a seismic shooting area off the Norwegian west coast. Fisheries Research 67: 143-150.

Smallwood, K.S., Rugge, L., Morrison, M.L., 2009. Influence of behavior on bird mortality in wind energy developments. Journal of Wildlife Management 73: 1082-1098.

Snohomish County Public Utility District No. 1., 2009. Draft Environmental Report. Admiralty Inlet Pilot Tidal Project. FERC Project No. 12690.

Snohomish PUD (Public Utility District), 2009. Admiralty Inlet Pilot Tidal Project, Appendix B: Aquatic species and project operations monitoring plan. Snohomish County Public Utility District. 14 pp.

Snyder, B., Kaiser, M.J., 2009. A comparison of offshore wind power development in Europe and the US: patterns and drivers of development. Applied Ecology 86: 1845-1856.

Stanley, D.R., Wilson, C.A., 2003. Seasonal and spatial variation in the biomass and size frequency distribution of fish associated with oil and gas platforms in the Northern Gulf of Mexico. In: Fisheries, Reefs, and Offshore Development. Stanley, D.R., Scarborough-Bull, A. (Eds.), American Fisheries Society Symposium 36, Bethesda, Maryland.

Stemler, J., 2007. Wind power siting regulations and wildlife guidelines in the United States. Unpublished report to U.S. Fish and Wildlife Service.

Stevenson, D., Chiarella, L., Stephan, D., Reid, R., Wilhelm, K., McCarthy, J., Pentony, M., 2004. Characterization of the Fishing Practices and Marine Benthic Ecosystems of the Northeast U.S. Shelf, and an Evaluation of the

Potential Effects of Fishing on Essential Fish Habitat. NOAA Technical Memorandum NMFS-NE-181.
Stokesbury, K.D.E., Harris, B.P., 2006. Impact of limited short-term sea scallop fishery on epibenthic community of Georges Bank closed areas. Marine Ecology Progress Series 307: 85-100.
Tasker, M., Jones, P.H., Dixon, T., Blake, B.F., 1984. Counting seabirds at sea from ships: a review of methods employed and a suggestion for a standardized approach. Auk 101: 567-577.
Tasker, M.L., Hope-Jones, P., Blake, B.F., Dixon, T.J., Wallis, A.W., 1986. Seabirds associated with oil production platforms in the North Sea. Ringing and Migration 7: 7-14.
Taylor, B.L., Martinez, M., Gerrodette, T., Barlow, J., Hrovat, Y.N., 2007. Lessons from monitoring trends in abundance of marine mammals. Marine Mammal Science 23: 157-175.
Taylor, C., 2001. Remote acoustic habitat assessment techniques used to characterize the quality and extent of oyster bottom in the Chesapeake Bay. Marine Geodesy 24: 171-189.
Taylor, P.D., Brzustowksi, J.M., Matkovich, C., Peckford, M.L., Wilson, D., 2010. radR: an open-source platform for acquiring and analyzing data on biological targets observed by surveillance radar. BiomedCentral 10: 22.
Teilmann, J., Tougaard, J., Carstensen, J., 2006a. Summary on harbour porpoise monitoring 1999-2006 around Nysted and Horns Rev Offshore Wind Farms. Ministry of the Environment, Copenhagen, Denmark.
Teilmann, J., Tougaard, J., Carstensen, J., Dietz, R., Tougaard, S., 2006b. Summary on seal monitoring 1999-2006 around Nysted and Horns Rev Offshore Wind Farms. Ministry of the Environment, Copenhagen, Denmark.
Teilmann, J., Tougaard, J., Carstensen, J., 2008. Effects from offshore wind farms on harbor porpoises in Denmark. In: Evans, P.G.H. (Ed.), Proceedings of the ASCOBANS/ECS Workshop; Offshore Wind Farms and Marine Mammals: Impacts and Methodologies for Assessing Impacts. ECS Special Publication Series No. 49, European Cetacean Society, pp. 50-59.
Thaxter, C.B., Burton, N.H.K., 2009. High definition imagery for surveying seabirds and marine mammals: A review of recent trials and development protocols. COWRIE Ltd., Norfolk, United Kingdom.
Thompson, P.M., Lusseau, D., Barton, T., Simmons, D., Rusin, J., Bailey, H., 2010. Assessing the responses of coastal cetaceans to the construction of offshore wind turbines. Marine Pollution Bulletin 60: 1200-1208.
Thomsen, F., Lüdemann, K., Kafemann, R., Piper, W., 2006. Effects of offshore wind farm noise on marine mammals and fish. COWRIE, The Crown Estate, London, U.K.

Thomsen, F., McCully, S.R., Weiss, L.R., Wood, D.T., Warr, K.J., Barry, J., Law, R.J., 2011. Cetacean stock assessments in relation to exploration and production industry activity and other human pressures: review and data needs. Aquatic Mammals 37: 1-93.

Thorne, R.E. ,1994. Hydroacoustic remote sensing for artificial habitats. Bulletin of Marine Science 55(3): 897-901.

Tougaard, J., Carstensen, J., 2011. Porpoises North of Sprogø Before, During and After Construction of and Offshore Wind Farm. National Environmental Research Institute, Aarhus University, Aarhus, Denmark. 45 pp.

Tougaard, J., Ebbesen, I., Tougaard, S., Jensen, T., Teilmann, J., 2003. Satellite tracking of harbour seals on Horns Reef; Use of the Horns Reef wind farm area and the North Sea. Biological Papers No. 3, Fisheries and Maritime Museum, Esbjerg, Denmark.

Tougaard, J., Carstensen, J., Teilmann, J., 2009a. Pile driving zone of responsiveness extends beyond 20 km for harbour porpoises (Phocoenaphocoena (L.). Journal of the Acoustical Society of America 126: 11-14.

Tougaard, J., Carstensen, J., Teilmann, J., Bech, N.I., 2005. Effects of the Nysted offshore wind farm on harbor porpoises. NERI Technical Report, Ministry of the Environment, Denmark.

Tougaard, J., Henriksen, O.D., Miller, L.A., 2009b. Underwater noise from three types of offshore wind turbines: estimation of impact zones for harbor porpoises and harbor seals. Journal of the Acoustical Society of America 125: 3766-3773.

Turner, A., Barrett, M., Brown, M.T., 2009. Processing of antifouling paint particles by Mytilus edulis. Environmental Pollution 157: 215-220.

U.S. Coast Guard, 2006. Neptune LNG Deepwater Port License Application: Final Environmental Impact Statement.

U.S. Navy, 2007. Final supplemental environmental impact statement for surveillance towed array sensor system low frequency active (SURTASS LFA) sonar. U.S. Navy, Arlington, VA.

USFWS (U.S. Fish and Wildlife Service), 2011. Examples of state and international guidance for duration and intensity of pre-construction and post-construction monitoring. Accessed online at http://www.fws.gov/windenergy/docs/Examples_State_International_Guidance_Duration_Intensity_Pre.pdf

Van den Berg, G.P., 2004. Effects of the wind profile at night on wind turbine sound. Journal of Sound and Vibration 227: 955-970.

Van den Berg, G.P., 2005. The beat is getting stronger: the effect of atmospheric stability on low frequency modulated sound of wind turbines. Journal of

Low Frequency Noise, Vibration And Active Control 24: 1-24
van der Wal, D., Forster, R.M., Rossi, F., Hummel, H., Ysebaert, T., Roose, F., Herman, P.M.J., 2011. Ecological evaluation of an experimental beneficial use scheme for dredged sediment disposal in shallow tidal waters. Marine Pollution Bulletin 62: 99-108.
van Hoey, G., Borja, A., Birchenough, S., Buhl-Mortensen, L., Degraer, S., Fleischer, D., Kerckhof, F., Magni, P., Muxika, I., Reiss, H., Schröder, A., Zettler, M.L., 2010. The use of benthic indicators in Europe: from the Water Framework Directive to the Marine Strategy Framework Directive. Marine Pollution Bulletin 60: 2187-96.
Verdant Power, 2010. Roosevelt Island Tidal Energy Project, FERC No. 12611, Final Kinetic Hydropower Pilot Licence Application, Volume 4, Part 1: Proposed Monitoring Plans, RITE Monitoring of Environmental Effects (RMEE). Verdant Energy, LLC, New York, NY.D144+D167 http://www.theriteproject.com/uploads/VPRITE-FLAVol4-Part1of3.pdf
Verfaillie, E., Degraer, S., Schelfault, K., Willems, W., Van Lancker, V., 2009. A protocol for classifying ecologically relevant marine zones, a statistical approach. Estuarine, Coastal and Shelf Science 83: 175-185.
Verhoef, J.P., Eecen, P.J., Nijdam, R.J., Korterink. H., Scholtens, H.H., 2004. WT-Bird: a Low Cost Solution for Detecting Bird Collisions. Petten, The Netherlands: ECN Wind Energy.
Von Oettingen, S., 2011. Bats and wind energy impacts. Presentation at conference, Orono, ME http://joomla.wildlife.org/Maine/images/WindEnergy/1_vonoettingen_2011%20tws%20wind%20conf%20svo2.pdf
Votier, S.C., Hatchwell, B.J., Beckerman, A., McCleery, R.H., Hunter, F.M., Pellatt, J., Trinder, M., Birkhead, T.R., 2005. Oil pollution and climate have wide-scale impacts on seabird demographics. Ecology Letters 8: 1157-1164.
Wahlberg, M., Westerberg, H., 2005. Hearing in fish and their reactions to sounds from offshore wind farms. Marine Ecology Progress Series 288: 295-309.
Walker, R., Weiss, L., Frojan, C.B., Basteri, D., Coggan, R., 2009. Strategic Review of Offshore Wind Farm Monitoring Data Associated with FEPA Licence Conditions, Benthic Ecology. Contract ME1117. CEFAS, Suffolk, UK. 19 pp.
Ware, S., Bolam, S.G., Rees, Hubert L, 2010. Impact and recovery associated with the deposition of capital dredgings at UK disposal sites: lessons for future licensing and monitoring. Marine Pollution Bulletin 60: 79-90.
Waring, G.T., Josephson, E., Maze-Foley, K., Rosel, P.E. (Eds.), 2009. U.S. Atlantic and Gulf of Mexico Marine Mammal Stock Assessments—2009. NOAA Technical Memorandum NMFS-NE-213. National Marine Fisheries Service, Woods Hole, MA.

Westerberg, H., Lagenfelt, I., 2008. Sub-sea Power Cables and the Migration Behaviour of the European Eel. Fisheries Management and Ecology 15(5-6): 369-375.

Wiese, F.K. Montevecchi, W.A., 2000. Marine bird and mammal surveys on the Newfoundland Grand Banks from offshore supply boats 1999-2000. Unpublished Husky Oil Contract Report, St. John's, Newfoundland.

Wiese, F.K., Montevecchi, W.A., Davoren, G.K., Huettmann, F., Diamond, A.W., Link, J., 2001. Seabirds at risk around offshore oil platforms in the North-west Atlantic. Marine Pollution Bulletin 42: 1285-1290.

Wiley, D.N., Moller, J.C., Zilinskas, K.A., 2003. The distribution and density of commercial fisheries and baleen whales within the Stellwagen Bank National Marine Sanctuary: July 2001-June 2002. MTS Journal 37 (1): 35-53.

Wilhelmsson, D., Malm, T., Thompson, R., Tchou, J., Sarantakos, G., McCormick, N., Luitjens, S., Gullström, M., Patterson Edwards, J.K., Amir, O., Dubi, A. (Eds), 2010. Greening Blue Energy: Identifying and Managing the Biodiversity Risks and Opportunities of Offshore Renewable Energy. International Union for the Conservation of Nature, Gland, Switzerland: IUCN, 102 pp.

Wilhelmsson, D., Malm, T., Ohman, M., 2006. The influence of offshore windpower on demersal fish. ICES Journal of Marine Science 63: 775-78.

Wilhelmsson, D., Malm, T., 2008. Fouling assemblages on offshore wind power plants and adjacent substrata. Estuarine, Coastal, and Shelf Science 79: 459-466.

Wilson, C.A., Miller, M.W., Allen, Y.C., Boswell, K.M., Nieland, D.L., 2006. Effects of depth, location, and habitat type on relative abundance and species composition of fishes associated with petroleum platforms and Sonnier Bank in the Northern Gulf of Mexico. U.S. Minerals Management Service.

Wilson, J.C., 2007. Offshore Wind Farms: Their Impacts, and Potential Habitat Gains as Artificial Reefs, in Particular for Fish. M.S. thesis, University of Hull, Hull, UK.

Wiltschko, W., Wiltschko, R., 2005. Magnetic orientation and magnetoreception in birds and other animals. Journal of Comparative Physiology A – Neuroethology and Sensory Neural and Behavioral Physiology 191: 675–693

Winkelman, J.E. 1992. The impact of the Sep wind park near Oosterbierum (Fr.), the Netherlands, on birds, 1: collision victims. RIN rapport 92/2. Arnhem IBN-DLO. (Dutch with English summary).

Zaugg, S., Saporta, G., van Loon, E., Schmaljohann, H., Liechti, R., 2008. Automatic identification of bird targets with radar via patterns produced by wing flapping. J.R. Soc. Interface 5(26): 1041-1053.

Zrnic, D.S., Ryzhkov, A.V., 1998. Observations of insects and birds with a polarimetric radar. IEEE Trans. Geosci. Rem Sens. 36: 661-668.

Zydlewski, G., McCleave, J., Viehman, H., Harmon, K., 2010. An initial assessment of fish presence and vertical distribution at two sites (control and proposed tidal power site) in Cobscook Bay. University of Maine, School of Marine Sciences, Orono, ME, and Maine Maritime Academy, Corning School of Ocean Studies, Castine, ME.

Appendix B. Offshore renewable energy impact matrix.

Level of Effect

Level of Certainty		Negligible/ Minor	Moderate	Major
	low			
	medium			
	high			

Level of Effect	Definition
**Negligible**	• No measurable effect.
**Minor**	• Should not influence or have only small effects on the affected resource, activity, or community.
**Moderate**	• Effects could moderately influence the resource, activity, or community, generally or for particular species.
**Major**	• Effects could significantly influence the resource, activity, or community, generally or for particular species.

Level of Certainty	Definition
**Low**	• Limited to no documentation or anecdotal evidence available
**Medium**	• Some documentation or anecdotal evidence available; no clear consensus among experts or within literature
**High**	•Well documented; consensus among experts and within the literature

Benthic Habitat and Resources Scale 1

Scale 1- Individual Device/ Demonstration Project			**Technology Type**												
			**Wind**				**Currents**				**Waves**				
			Foundation				***Turbine Type***				***Device Type***				
							**Open Rotor**		**Shrouded**						
		**Potential Effect**	**Monopile**	**Gravity**	**Tripod/ lattice**	**Floating Mooring**	**Bottom Mounted**	**Floating Mooring**	**Bottom Mounted**	**Floating Mooring**	**Point Absorber**	**Wave Attenuator**	**Oscillating Water Column**	**Oscillating Wave Surge Converter**	**Overtopping**
Benthic Habitat and Resources	Physical	Changes to currents, wave regime													
		Disturbance from installation/removal device (including increased turbidity)													
		Disturbance from installation of power cable (including trenching)													
		Disturbance removal of power cable													
		Scour around structures													
		Smothering by excavated sediments													
		Reef effects													
		Increase in sediment temperature around cable													
	Chemical	Diffusion/flaking marine coating													
		Leakage lubricants/fluids;Release maintenance chemicals													
		Large spills or accidents													
		Chemicals discharged during installation or removal													
		Resuspension of pollutants in sediments													
	Acoustic	Noise and vibration before and during construction; during operation; during decomissioning													
	EMF	EMF from power cable													

Benthic Habitat and Resources Scale 2

Scale 2- Single Commercial Scale Facility			Technology Type												
			Wind				Currents				Waves				
			Foundation				*Turbine Type*				*Device Type*				
							Open Rotor		Shrouded						
		**Potential Effect**	Monopile	Gravity	Tripod/lattice	Floating Mooring	Bottom Mounted	Floating Mooring	Bottom Mounted	Floating Mooring	Point Absorber	Wave Attenuator	Oscillating Water Column	Oscillating Wave Surge Converter	Overtopping
**Benthic Habitat and Resources**	Physical	Changes to currents, wave regime													
		Disturbance from installation/removal device (including increased turbidity)													
		Disturbance from installation of power cable (including trenching)													
		Disturbance removal of power cable													
		Scour around structures													
		Smothering by excavated sediments													
		Reef effects													
		Increase in sediment temperature around cable													
	Chemical	Diffusion/flaking marine coating													
		Leakage lubricants/fluids; Release maintenance chemicals													
		Large spills or accidents													
		Chemicals discharged during installation or removal													
		Resuspension of pollutants in sediments													
	Acoustic	decommissioning													
	EMF	EMF from power cable													

Fish and Fisheries Resources Scale 1

Scale 1- Individual Device/ Demonstration Project			Technology Type												
			Wind				Currents				Waves				
			Foundation				*Turbine Type*				*Device Type*				
							Open Rotor		Shrouded						
		**Potential Effect**	Monopile	Gravity	Tripod/ lattice	Floating Mooring	Bottom Mounted	Floating Mooring	Bottom Mounted	Floating Mooring	Point Absorber	Wave Attenuator	Oscillating Water Column	Oscillating Wave Surge Converter	Overtopping
Fish	Physical	Disturbance from installation device													
		Disturbance from removal of device													
		Disturbance from installation of power cable													
		Disturbance removal of power cable													
	Dynamic	Collison/Blade Strike													
		Pressure gradients around rotor													
		Velocity gradients around rotor													
		Rotor wake													
	Chemical	Diffusion/flaking marine coating													
		Leakage lubricants/fluids during operation													
		Large spills or accidents													
		Chemicals discharged during installation or removal													
		Resuspension of pollutants in sediments													
	Acoustic	Operational noise (gears, mooring chains, etc.)													
		Noise from pre-construction seismic surveying													
		Noise from vessels conducting project maintenance/monitoring													
		Noise from pile driving													
		Noise from vessel traffic during installation													
		Noise from directional drilling for power cable													
		Noise from pile cutting during device removal													
		Noise from vessel traffic during removal													
	EMF	EMF from power cable													
	Community level impacts	Habitat alteration/community composition													
		Reef effects (aggregation)													
Fisheries		Decreased Catchability during construction													
		Decreased Catchability during operation													
		Loss of access to grounds during construction													
		Loss of access to grounds during operation - mobile fishing gear													
		Loss of access to grounds during operation - fixed and recreational fishing gear													
		Changes in species distribution													
		Reef effects (aggregation)													
		Damage/lost gear													

Fish and Fisheries Resources Scale 2

Scale 2- Single Commercial Scale Facility			Technology Type												
			**Wind**				**Currents**				**Waves**				
			Foundation				***Turbine Type***				***Device Type***				
							**Open Rotor**		**Shrouded**						
		**Potential Effect**	**Monopile**	**Gravity**	**Tripod/lattice**	**Floating Mooring**	**Bottom Mounted**	**Floating Mooring**	**Bottom Mounted**	**Floating Mooring**	**Point Absorber**	**Wave Attenuator**	**Oscillating Water Column**	**Oscillating Wave Surge Converter**	**Overtopping**
**Fish**	Physical	Disturbance from installation device													
		Disturbance from removal of device													
		Disturbance from installation of power cable													
		Disturbance removal of power cable													
	Dynamic	Collison/Blade Strike													
		Pressure gradients around rotor													
		Velocity gradients around rotor													
		Rotor wake													
	Chemical	Diffusion/flaking marine coating													
		Leakage lubricants/fluids during operations													
		Large spills or accidents													
		Chemicals discharged during installation or removal													
		Resuspension of pollutants in sediments													
	Acoustic	Operational noise (gears, mooring chains, etc.)													
		Noise from pre-construction seismic surveying													
		Noise from pile driving													
		Noise from vessel traffic during installation													
		Noise from directional drilling for power cable													
		Noise from pile cutting during device removal													
		Noise from vessel traffic during removal													
	EMF	EMF from power cable													
	Community level impacts	Habitat alteration/community composition													
		Reef effects (aggregation)													
**Fisheries**		Decreased Catchability during construction													
		Decreased Catchability during operation													
		Loss of access to grounds during construction													
		Loss of access to grounds during operation - mobile gear fisheries													
		Loss of access to grounds during operation - fixed and recreational fisheries													
		Changes in species distribution													
		Reef effects (aggregation)													
		Damage/lost gear													

Avian species Scale 1

Scale 1- Individual Device/ Demonstration Project			Technology Type														
			Wind				Currents				Waves				
			Foundation				*Turbine Type*				*Device Type*				
							Open Rotor		Shrouded						
		**Potential Effect**	**Monopile**	**Gravity**	**Tripod/ lattice**	**Floating Mooring**	**Bottom Mounted**	**Floating Mooring**	**Bottom Mounted**	**Floating Mooring**	**Point Absorber**	**Wave Attenuator**	**Oscillating Water Column**	**Oscillating Wave Surge Converter**	**Overtopping**
Avian Species	Physical	Displacement or attraction to structure above surface of the water													
		Displacement or attraction to structure below surface of the water													
		Disorientation or attraction from lighting													
		Disturbance from installation device or transmission cable													
		Disturbance from removal of device or transmission cable													
	Dynamic	Collision with rotating turbine blades													
		Pressure gradients around rotor													
		Velocity gradients around rotor													
		Rotor wake													
	Chemical	Diffusion/flaking marine coating													
		Leakage lubricants/fluids; Release maintenance chemicals													
		Large spills or accidents													
		Chemicals discharged during installation or removal													
		Resuspension of pollutants in sediments													
	Acoustic	Operational noise (rotor, power train, cable strum)													
		Noise from pile driving													
		Noise from vessel traffic during installation, maintanence and removal													
		Noise from directional drilling for power cable													
		Noise from pile cutting during device removal													
	EMF	EMF from power cable													
	Energy	Changes to foraging due to changes in turbulent dissipation/boundary layers													
		Changes to foraging due to changes to wave energy regime													

Avian species Scale 2

Scale 2- Single Commercial Scale Facility			Technology Type												
			Wind				Currents				Waves				
			Foundation				*Turbine Type*				*Device Type*				
							Open Rotor		Shrouded						
		Potential Effect	Monopile	Gravity	Tripod/lattice	Floating Mooring	Bottom Mounted	Floating Mooring	Bottom Mounted	Floating Mooring	Point Absorber	Wave Attenuator	Oscillating Water Column	Oscillating Wave Surge Converter	Overtopping
**Avian Species**	Physical	Displacement or attraction to structure above surface of the water													
		Displacement or attraction to structure below surface of the water													
		Disorientation or attraction from lighting													
		Disturbance from installation device or transmission cable													
		Disturbance from removal of device or transmission cable													
	Dynamic	Collision with rotating turbine blades													
		Pressure gradients around rotor													
		Velocity gradient around rotor													
		Rotor wake													
	Chemical	Diffusion/flaking marine coating													
		Leakage lubricants/fluids; Release maintenance chemicals													
		Large spills or accidents													
		Chemicals discharged during installation or removal													
		Resuspension of pollutants in sediments													
	Acoustic	Operational noise (rotor, power train, cable strum)													
		Noise from pile driving													
		Noise from vessel traffic during installation, maintanence and removal													
		Noise from directional drilling for power cable													
		Noise from pile cutting during device removal													
	EMF	EMF from power cable													
	Energy	Changes to foraging due to changes in turbulent dissipation/boundary layers													
		Changes to foraging due to changes to wave energy regime													

Marine mammals and sea turtles Scale 1

Scale 1- Individual Device/ Demonstration Project			Technology Type												
			Wind				Currents				Waves				
			Foundation				*Turbine Type*				*Device Type*				
							Open Rotor		Shrouded						
		Potential Effect	Monopile	Gravity	Tripod/ lattice	Floating Mooring	Bottom Mounted	Floating Mooring	Bottom Mounted	Floating Mooring	Point Absorber	Wave Attenuator	Oscillating Water Column	Oscillating Wave Surge Converter	Overtopping
Marine Mammals and Sea Turtles	Physical	Reef Effects													
		Englanglement with mooring lines or cables													
	Dynamic	Collision with or avoidance of rotating turbine blades													
		Strike by installation or support vessels													
	Chemical	Diffusion/flaking marine coating													
		Leakage lubricants/fluids;Release maintenance chemicals													
		Large spills or accidents													
		Chemicals discharged during installation or removal													
		Resuspension of pollutants in sediments													
	Acoustic	Operational noise (rotor, power train, cable strum)													
		Noise from pile driving													
		Noise from vessel traffic during installation or removal													
		Noise from directional drilling for power cable													
		Noise from pile cutting during device removal													
	EMF	EMF from power cable													

Marine mammals and sea turtles Scale 2

Scale 2- Single Commercial Scale Facility			Technology Type												
			Wind				Currents				Waves				
			Foundation				*Turbine Type*				*Device Type*				
							Open Rotor		Shrouded						
		Potential Effect	Monopile	Gravity	Tripod/lattice	Floating Mooring	Bottom Mounted	Floating Mooring	Bottom Mounted	Floating Mooring	Point Absorber	Wave Attenuator	Oscillating Water Column	Oscillating Wave Surge Converter	Overtopping
Marine Mammals and Sea Turtles	Physical	Reef Effects													
		Englanglement with mooring lines or cables													
	Dynamic	Collision with or avoidance of rotating turbine blades													
		Strikes with installation or support vessels													
	Chemical	Diffusion/flaking marine coating													
		Leakage lubricants/fluids;Release maintenance chemicals													
		Large spills or accidents													
		Chemicals discharged during installation or removal													
		Resuspension of pollutants in sediments													
	Acoustic	Operational noise (rotor, power train, cable strum)													
		Noise from pile driving													
		Noise from vessel traffic during installation or removal													
		Noise from directional drilling for power cable													
		Noise from pile cutting during device removal													
	EMF	EMF from power cable													
